# Synthesis and Pharmacological Characterization of Novel Peripheral Cannabinoid-1 Receptor Blockers Based on a Tricyclic Scaffold

**DOI:** 10.1021/acs.jmedchem.4c03132

**Published:** 2025-04-21

**Authors:** Asaad Gammal, Taher Nassar, Yael Soae, Noam Freeman, Amit Badihi, Saja Baraghithy, Alina Nemirovski, Joseph Tam, Simon Benita

**Affiliations:** † Obesity and Metabolism Laboratory, The Institute for Drug Research, School of Pharmacy, Faculty of Medicine, The Hebrew University of Jerusalem, Jerusalem 9112001, Israel; ‡ Laboratory of Nano Delivery Systems, The Institute for Drug Research, School of Pharmacy, Faculty of Medicine, The Hebrew University of Jerusalem, Jerusalem 9112001, Israel; § BioNanoSim (BNS), Hadassah Ein Kerem Campus, Minrav Building (JBP), Jerusalem 9112101, Israel

## Abstract

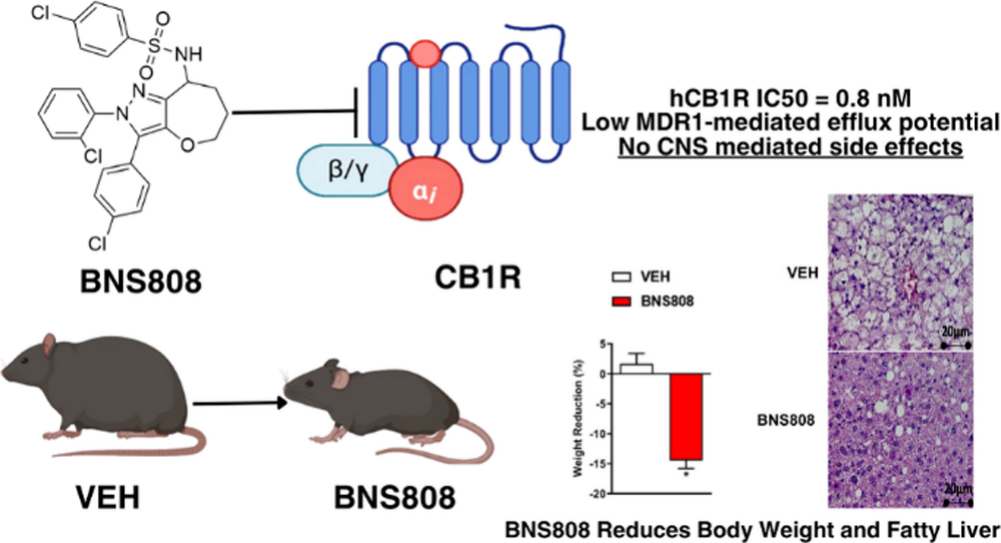

The development of peripherally selective cannabinoid-1 receptor (CB_1_R) antagonists offers a promising strategy for obesity treatment. Here, we evaluated the efficacy of novel tricyclic CB_1_R antagonists, focusing on BNS808. Our findings demonstrate that BNS808 exhibits robust CB_1_R antagonism with notable CB_2_R selectivity, minimal brain penetration, and potent in vitro and in vivo efficacy. The compound’s high plasma protein binding reduces free drug availability for CNS entry, enhancing safety and minimizing drug–drug interactions. In diet-induced obese mice, BNS808 effectively reduced body weight, adiposity, liver triglycerides, and liver enzymes, supporting its peripherally mediated action. These results highlight BNS808 as a promising candidate for obesity treatment. Additionally, our novel library of peripherally selective CB_1_R antagonists provides a strong foundation for future drug development. With further refinement, BNS808 holds significant clinical potential to address the global obesity epidemic.

## Introduction

The global prevalence of obesity has markedly risen over the past three decades.^1^ Obesity is linked with numerous complications, including a direct impact on liver function, both independently and in conjunction with other underlying factors.^2^ Notably, metabolic dysfunction-associated steatotic liver disease (MASLD) constitutes a substantial contributor to morbidity and mortality in Western societies.^2^ The U.S. Food and Drug Administration (FDA) and the European Medicines Agency (EMA) have sanctioned any medications explicitly for treating MASLD.^3−5^ However, Madrigal Pharmaceuticals’ resmetirom, recently approved for metabolic dysfunction-associated steatohepatitis (MASH), offers promise. However, its limitations, including potential off-target effects and long-term treatment, underscore the necessity for alternative therapeutic strategies.^6−9^

Recent studies have emphasized the involvement of the endocannabinoid system (ECS) and the cannabinoid-1 receptor (CB1R) activation in developing obesity and its associated metabolic complications, such as MASLD.^10,11^ Given that endocannabinoids (eCBs) are found in the liver at levels comparable to those in the brain,^12,13^ it was reasonable to hypothesize that the ECS/CB_1_R system plays a significant role in regulating lipid metabolism in the liver.^10^ Indeed, in cases of MASLD, various signaling pathways in hepatocytes appear to be influenced by ECS modulation.^14−19^ One notable pathway involves the activation of CB_1_R in rodents, which has been demonstrated to influence the hepatic rate of de novo lipogenesis.^20,21^ A high-fat diet (HFD) has also been observed to increase hepatic eCB production^15,22,23^ and CB_1_R expression,^19^ leading to elevated eCB “tone” that suppresses mitochondrial free fatty acids (FFAs) β-oxidation.^24,25^

Consequently, disrupting ECS activity through CB_1_R blockade has been proposed as a potential approach to mitigate obesity-induced hepatic steatosis and enhance systemic metabolism.^11,15−17,19,20^ This concept prompted pharmaceutical companies to develop CB_1_R-blocking drugs as possible treatments for obesity and its associated comorbidities. One such compound, rimonabant (Acomplia),^26^ demonstrated promise by effectively reducing body weight in obese and overweight individuals and improving metabolic abnormalities,^14,27−29^ including MASLD.^30^ However, its usage was hindered by neuropsychiatric side effects, such as depression, anxiety, and suicidal ideation, leading to its worldwide withdrawal from the market in 2009.^31,32^ Other structurally distinct CB_1_R antagonists (e.g., otenabant, taranabant, and ibipinabant) also progressed to late-stage clinical testing but exhibited similar CNS side effect profiles,^33,34^ rendering them unsuitable as valid therapeutics.

Recent genomic data suggest that specific polymorphisms in the CB_1_R gene, either independently or in conjunction with variants in the serotonin transporter gene, are linked to the onset of anxiety and depression.^35−37^ These findings suggest utilizing genetic screening to identify a patient cohort that could benefit from CB_1_R antagonist treatment with a more favorable safety profile.^38^ However, until such screening methods are established, an alternative strategy would involve harnessing the therapeutic potential of CB_1_R inhibition while circumventing the adverse effects associated with its CNS impact.^39−41^ This could be achieved by targeting CB_1_Rs in lower but functionally significant levels in various peripheral tissues, including the liver.^19^

To pursue this strategy, multiple research groups, including ours, have been actively engaged in this area in recent years.^42−48^ The basic approach involves modifying various physiochemical properties^49,50^ to limit brain exposure of globally acting CB_1_R blockers, adhering to the Lipinski rule of five^51^ for non-blood brain barrier (BBB) permeable drugs. For instance, this can be achieved by increasing the calculated relative lipophilicity (cLogP > 5), molecular weight (*M*_W_ > 500 g/mol), polar surface area (PSA > 60 Å), and hydrogen bonding donor capacity (HBD > 5).^52−55^ Additionally, these molecules should exhibit limited brain penetration, partly due to recognition by CNS efflux transporters, thereby reducing the risk of CNS-mediated side effects.^51^

**Scheme 1:**
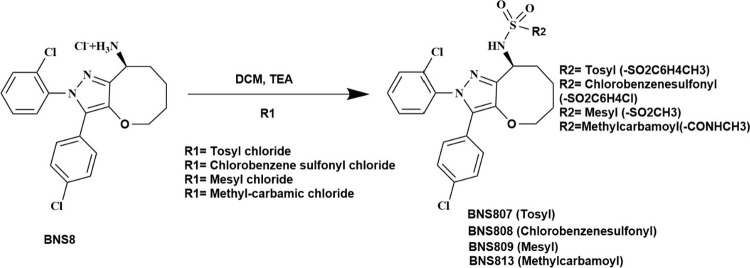
Chemical Synthesis of BNS807, BNS808, BNS809, and BNS813, According to General Procedure A, as Described in the Experimental Section Representation of synthetic transformation where R1 denotes the reactant functional group (tosyl chloride, Chlorobenzene sulfonyl chloride, mesyl chloride, or methyl-carbamic chloride), and R2 represents the final substituent (−SO_2_C_6_H_4_CH_3_, −SO_2_C_6_H_4_Cl, −SO_2_CH_3_, or −CONHCH_3_) incorporated into the product.

**Scheme 2:**
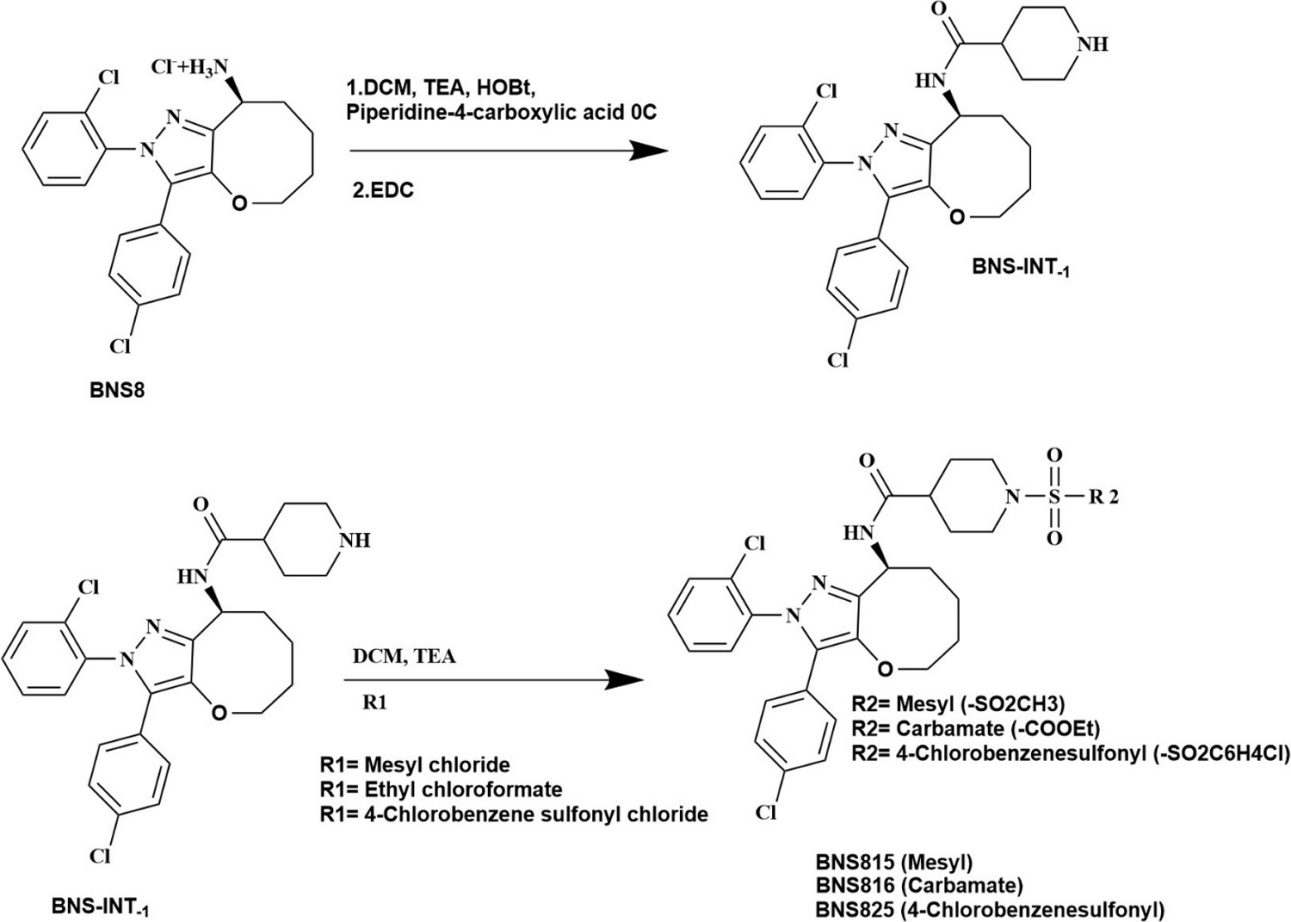
Chemical Synthesis of BNS815, BNS816, and BNS825, According to General Procedure B, as Described in the Experimental Section Representation of synthetic transformation where R1 denotes the reactant functional group (Mesyl chloride, Ethyl chloroformate, or 4-Chlorobenzene sulfonyl chloride), and R2 represents the final substituent (−SO_2_CH_3_, −COOEt, or −SO_2_C_6_H_4_Cl) incorporated into the product.

In the present study, we synthesized and assessed novel CB_1_R blockers by chemically attaching different moieties to the molecular building block (BNS8),^56^ as depicted in Schemes 1 and 2. Subsequently, their physicochemical properties were calculated, and their affinity and selectivity for murine and human CB_1_R and CB_2_R were determined. Furthermore, upon identification of the lead compound, assessments were conducted on its non-penetration in the brain, potency, biodistribution, and efficacy in obesity and its associated metabolic abnormalities.

## Results

### Compound Synthesis

In this study, we synthesized a select group of tricyclic CB_1_R antagonists (BNS compounds) to evaluate the impact of structural modifications on peripheral CB_1_R selectivity and pharmacokinetic properties. The compounds were designed to retain the core tricyclic scaffold, known for its CB_1_R antagonism, while introducing minor modifications to reduce CNS penetration and improve peripheral selectivity. Structural changes were made to optimize reduced BBB permeability, ensuring prolonged peripheral activity. The novel compounds were synthesized from the scaffold of BNS8 according to procedures A and B described in the methods and Schemes 1 and 2.

### Binding and Activity Profiles of the Compounds

**Scheme 3:**
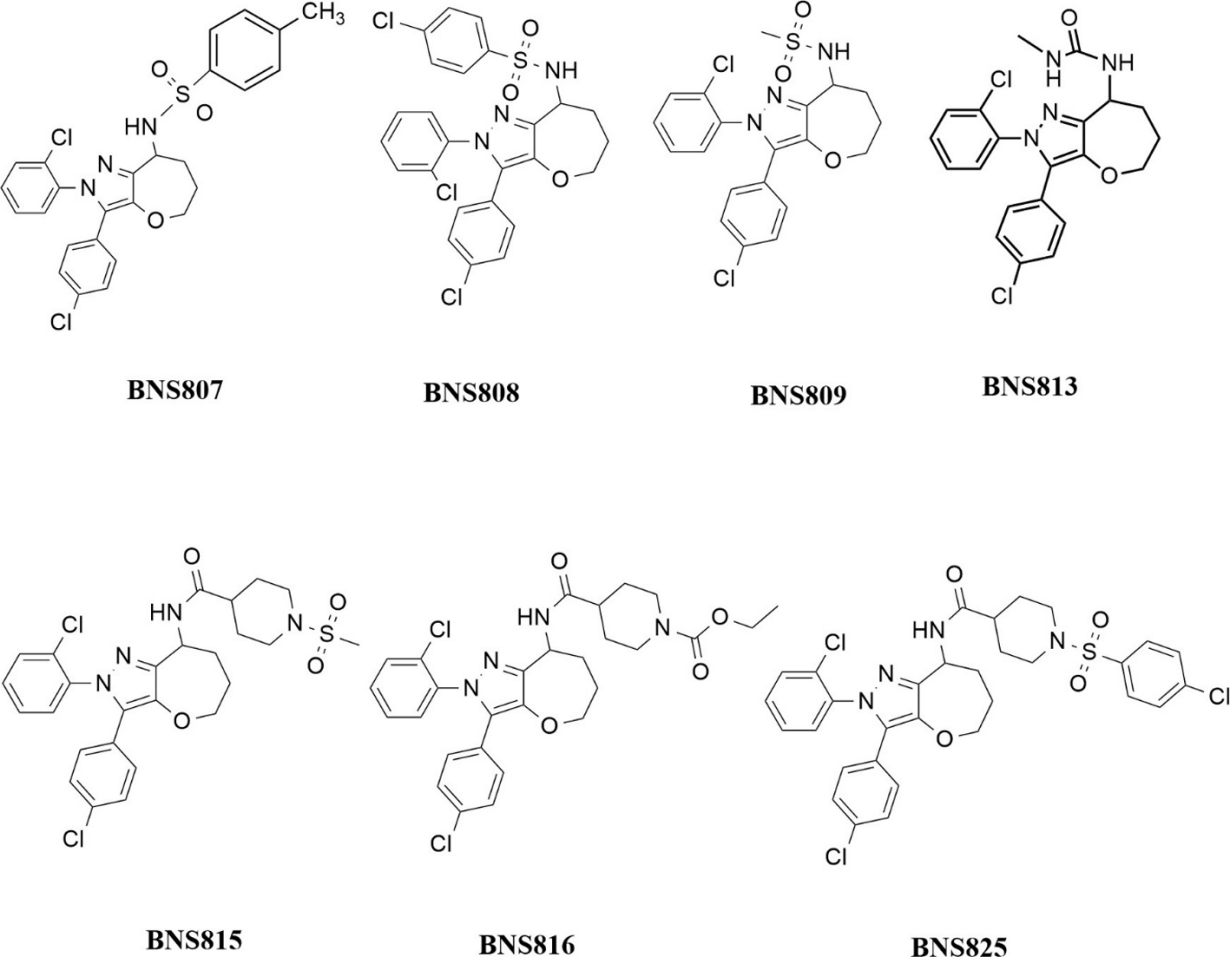
Chemical Structures of all the Tested Compounds

The synthesized compounds (Scheme 3) were evaluated for their binding affinity (*K*_i_) and bioactivity (IC_50_) to mouse and human CB_1_R (Tables 1 and 2). Among them, BNS808, generated from chlorobenzene sulfonamide, showed low *K*_i_ values of 0.7 nM for both mouse and human CB_1_R (with a hIC_50_ of 0.8 nM). Similarly, compounds derived from 4-methyl benzenesulfonamide (BNS807), ethyl 4-carbamoylpiperidine-1-carboxylate (BNS816), and 1-(4-chlorophenyl) sulfonyl-piperidine-4-carboxamide (BNS825) exhibited m*K*_i_ values of 10, 8.9, and 1.6 nM, respectively, indicating their strong binding affinity to CB_1_R.

**Table 1:** Physicochemical Properties of the Novel Derivative Compounds Based on Building Block BNS8^a^

compound ID#	*M*_W_ (g/mol)	cLogP	PSA (A^2^)	HBD	CB_1_R			CB_2_R	*P*_app_A: B (× 10^–6^ cm/s)	efflux ratio	P-gp substrate
					m*K*_i_ (nM)	h*K*_i_ (nM)	hIC_50_ (nM)	h*K*_i_ (nM)			
BNS807	528	6.58	73	1	10	1.4	1.5	1650	ND	ND	ND
BNS808	549	6.81	73	1	0.7	0.7	0.8	1930	0.26	3.95	yes
BNS809	452	4.6	73	1	33.78	7.7	8.5	>9000	ND	ND	ND
BNS813	431	4.87	68	2	1130	ND	ND	ND	ND	ND	ND

a ND = not determined.

**Table 2:** Physicochemical Properties of the Novel Derivative Compounds Based on Building Block BNS8-INT-1^a^

compound ID#	*M*_W_ (g/mol)	cLogP	PSA (A^2^)	HBD	CB_1_R			CB_2_R	*P*_app_A: B (×10^–6^ cm/s)	efflux ratio	P-gp substrate
					m*K*_i_ (nM)	h*K*_i_ (nM)	hIC_50_ (nM)	h*K*_i_ (nM)			
BNS815	563	4.72	93	1	17.96	9.09	10.09	6312	10.09	1.87	yes
BNS816	557	5.80	86	1	8.93	ND	ND	1531	6.99	0.77	no
BNS825	660	6.93	93	1	1.6	3.1	3.8	127	ND	ND	ND

a ND = not determined.

Additionally, all novel compounds demonstrated a CB_1_R antagonistic profile, with only BNS807 exhibiting an inverse agonistic profile. At the same time, all the other were found to be neutral antagonists, as measured by the GTPγS assay (Supplementary Figure 1). Moreover, most compounds showed negligible binding affinity to the CB_2_R, indicating selectivity toward CB_1_R.

P-gp acts as a gatekeeper in the brain, preventing the entry of foreign substances and returning them to the blood. Recent studies have designed and tested multiple molecules with peripheral selectivity toward CB_1_R, aiming for limited BBB penetrations.^39^ Many of these molecules possess similar characteristics, being less hydrophobic and more polar to hinder brain penetrance and diffusion across the BBB. Therefore, we next assessed the potential of the novel BNS8 compounds to cross the BBB by conducting a bi-directional permeability test across MDR1-MDCKII cells. The results, summarized in Tables 1, 2 and Supplementary Table 2, report each compound’s mean Papp and efflux ratio. Notably, BNS808 was identified as a P-gp substrate, a desirable characteristic for peripherally restricted CB_1_R blockers.

### Cell Viability and the Mutagenic Effect of BNS8 Compounds

Given the liver’s pivotal role in metabolizing and eliminating chemicals, we next employed HepG2 cells to assess the cytotoxicity of BNS808 and other hit compounds, as presented in Table 3 and Supplementary Table 5. Staurosporine, a reference toxic compound, exhibited cytotoxicity with an IC_50_ of 0.06 μM in HepG2 cells. Among the tested compounds, BNS807, BNS808, BNS815, and BNS825 displayed IC_50_ values of 17.95, 16.84, 46.69 μM, and above 100 μM, respectively.

**Table 3:** Summary of In Vitro Assays^a^

ID#	stability in SGF and SIF^b^	cell viability relative IC_50_ (μM)^b^	hERG assay IC_50_ (μM)^b^	protein binding in mouse brain^b^	mini-Ames assay^b^
BNS807	ND	17.95	18.04	ND	ND
BNS808	stable	16.84	5.39	high	nonmutagenic^b^
BNS815	ND	46.69	4.86	ND	ND
BNS825	ND	>100	ND	ND	ND

a ND = not determined.

b Detailed Results in the Supplementary Tables 3–9.

Considering that BNS808 had the lowest CB_1_R *K*_i_ value, dividing its cell viability IC_50_ (16.84 μM) by its CB_1_R binding value (either *K*_i_ 0.7 nM or IC_50_ 0.8 nM) suggests very low cytotoxicity in hepatocytes, if any. Therefore, its mutagenic potential was further investigated using the mini-Ames assay, demonstrating no toxicity at doses ranging from 31.25 to 1000 μg/well on the two bacteria strains (TA98, TA100). Moreover, a more than 2-fold increase in reversion over the negative control was measured, indicating that this compound did not induce substantial dose-dependent increases in reversion rates on the two tested strains, both with and without S9 (Table 3 and Supplementary Tables 3, 4). These findings suggest that the parent compound, as well as its metabolites, are nonmutagenic.

### Evaluation of the Cardiotoxicity of BNS8 Compounds

The cardiotoxicity evaluation of the novel compounds considered the potential inhibition of the human ether-à-go-go-related gene (hERG) cardiac potassium channel, which can lead to cardiac arrhythmias and drug development failure. The automated patch clamp method (SyncroPatch 384PE) was employed for this assessment. According to the results presented in Table 3 and Supplementary Table 6, BNS808 demonstrated a hERG IC_50_ of 5.39 μM, suggesting low potential for cardiotoxicity (QT prolongation) since its IC_50_ value for CB_1_R is 0.8 nM and the acceptance criteria for the hERG assay are a hERG IC_50_ > 100 × CB_1_R IC_50_.

### CYP Inhibition of BNS808 in Human Liver Microsomes

The evaluation of BNS808’s ability to inhibit cytochrome P450 (CYP) enzyme was conducted using human liver microsomes, which contain a variety of drug-metabolizing enzymes, including CYP1A2, CYP2C9, CYP2C19, CYP2D6, and CYP3A4. Interestingly, BNS808 exhibited moderate inhibition of CYP3A4, weak-moderate inhibition of CYP2C9 and CYP2C19, and weak inhibition of CYP1A2 and CYP2D6, with IC_50_ values of 2.99, 8.33, 18.0, and >50 μM, respectively.

### Protein Binding Efficacy of BNS808

The protein binding of BNS808 was assessed using the equilibrium dialysis assay to evaluate its potential for peripheral retention and minimal brain penetration. The results indicate that BNS808 exhibits extremely high protein binding in mouse brain tissue, with no detectable free drug observed in the receiver compartment (Table 3 and Supplementary Table 9). This finding supports the hypothesis that BNS808 is predominantly bound to proteins, limiting its free availability to block CB_1_R in the CNS. Such properties are consistent with its design as a peripherally selective CB_1_R antagonist.

### Reduced Brain Penetration of BNS808

**Figure 1:**
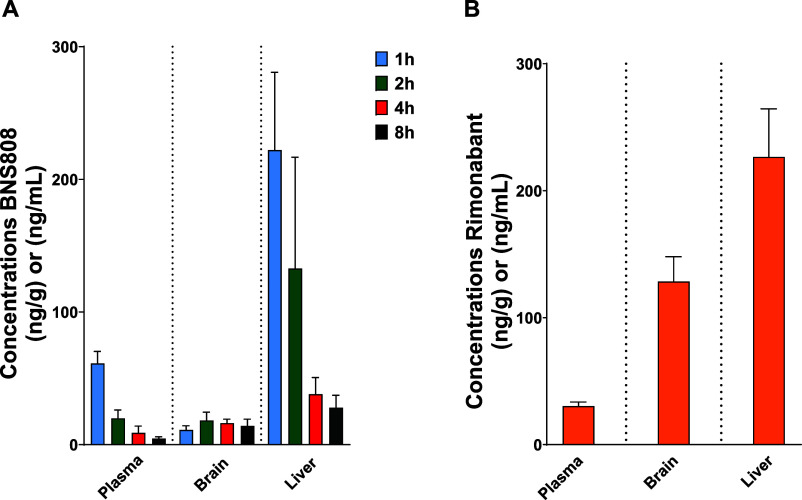
Biodistribution of BNS808 and rimonabant in plasma, brain, and liver following an acute oral administration. The accumulation of BNS808 (A) and rimonabant (B) in organs/plasma was evaluated by analyzing the drug levels in the plasma, brain, and liver for 1, 2, 4, and 8 h (for BNS808) and 1-h (for rimonabant) post-acute administration of 1 mg/kg PO to lean C57Bl/6 mice. Data represent the mean ± SEM of a minimum of 3 mice for each group.

The brain-to-plasma (B/P) concentration ratio, a parameter used to estimate CNS pharmacokinetics and BBB availability of compounds, was assessed in vivo. This ratio indicates the concentration of the free form of the drug in the brain, which is responsible for eliciting the relevant pharmacological response at the target site. Following acute or chronic oral administration of 1 mg/kg BNS808 to mice, we measured the compound’s brain, plasma, and liver concentrations. Consistent with the in vitro permeability results, BNS808, with its low permeability and classification as a P-gp substrate, demonstrated limited brain penetrance (an average of 13.7 ng/g) after a single acute dose at different time points (Figure 1A) and a similar concentration (∼20 ng/g) after chronic treatment (Supplementary Figure 2), with a B/P ratio of 0.18 at 1 h post-acute administration, primarily due fast clearance from the plasma and therefore low circulating levels. Interestingly, the compound showed higher concentrations in the liver (∼200 ng/g), resulting in a liver/plasma (L/P) ratio of 3.62 at 1 h post-acute administration. In contrast, rimonabant (administered at the same dose of 1 mg/kg) exhibited concentrations of 128 ng/g in the brain, 226 ng/g in the liver, and 30 ng/mL in the plasma 1 h post-acute administration, resulting in B/P ratio of 4.28 and L/P ratio of 7.4 (Figure 1B).

### BNS808 Does Not Induce CNS-Mediated Side Effects

**Figure 2:**
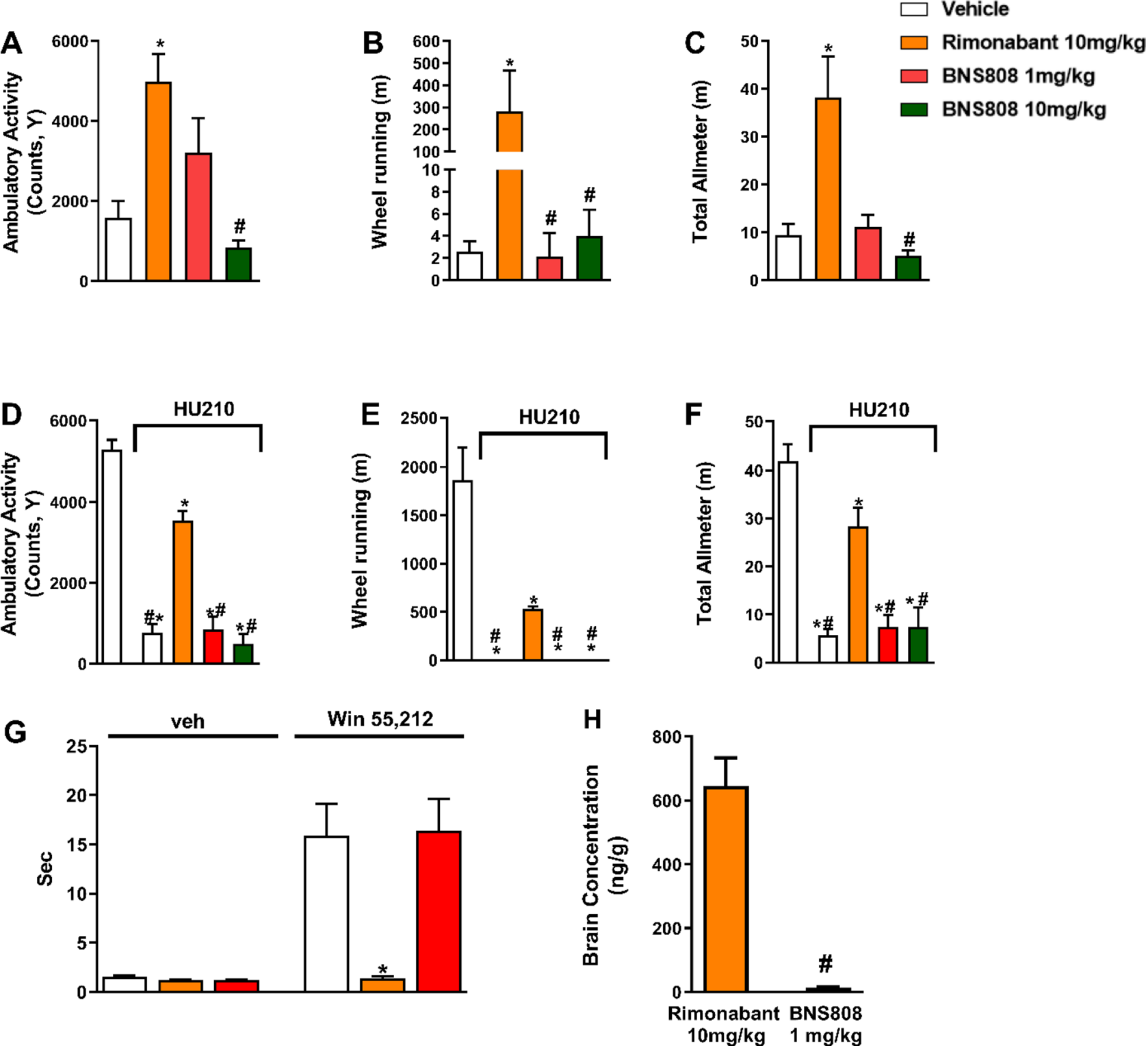
BNS808 does not induce CNS-mediated side effects. The ability of BNS808 and rimonabant (as a positive control) to induce centrally mediated hyperactivity in mice (A–C). Wild-type, male, C57Bl/6J mice received a single dose of rimonabant (10 mg/kg, PO), BNS808 (1 or 10 mg/kg, PO) or vehicle, and the Promethion High-Definition Behavioral Phenotyping System monitored the activity profile. In addition, the ability of BNS808 and rimonabant (as a positive control) to inhibit the hypomotility induced by a CB_1_R receptor agonist (HU210; 30 μg/kg, IP) was tested in wild-type, male, C57Bl/6J mice which received a single dose of rimonabant (10 mg/kg, PO) or BNS808 (1 or 10 mg/kg, PO) or vehicle (D–F). Only Rimonabant (10 mg/kg, PO) blocked the cataleptic behavior mediated by WIN-55,212 (G). All these results were coupled by measuring the compound levels in the brain, demonstrating that the brain’s rimonabant levels were 600 ng/g, whereas BNS808 levels were 13 ng/g after 1 h of PO administration (H). Data represent the mean ± SEM from 4 to 8 mice per group. *P < 0.05 vs Vehicle-treated control. ^#^*P* < 0.05 vs rimonabant.

BNS808 was further evaluated for its potential to induce CNS-mediated side effects, and the results are presented in Figure 2. In contrast to rimonabant, known to penetrate the brain and cause CNS-mediated side effects^57^ our study demonstrates promising outcomes for BNS808. No CNS-mediated side effects were observed in mice at low (1 mg/kg, PO) and high (10 mg/kg, PO) doses of BNS808. Specifically, BNS808 exhibited no significant changes in locomotor activity compared to rimonabant (Figure 2A–C) and was unable to antagonize the hypoactivity mediated by HU-210, a CB_1_R agonist, given 30 min before administrating BNS808 (Figure 2D–F).

We next assessed the ability of BNS808 to antagonize the brain’s CB_1_R-induced catalepsy and found that only rimonabant (10 mg/kg, PO) blocked the cataleptic behavior mediated by the CB_1_R agonist WIN-55,212 (Figure 2G). All these results were validated by the levels of each drug in the brain, as shown in Figure 2H. Unlike rimonabant, the lack of CNS-mediated side effects observed with BNS808 is attributed to its lower brain penetrance, which further highlights its potential advantage over rimonabant in terms of its safety profile.

### BNS808 Improves Metabolic Profile in Diet-Induced Obese Mice

**Figure 3:**
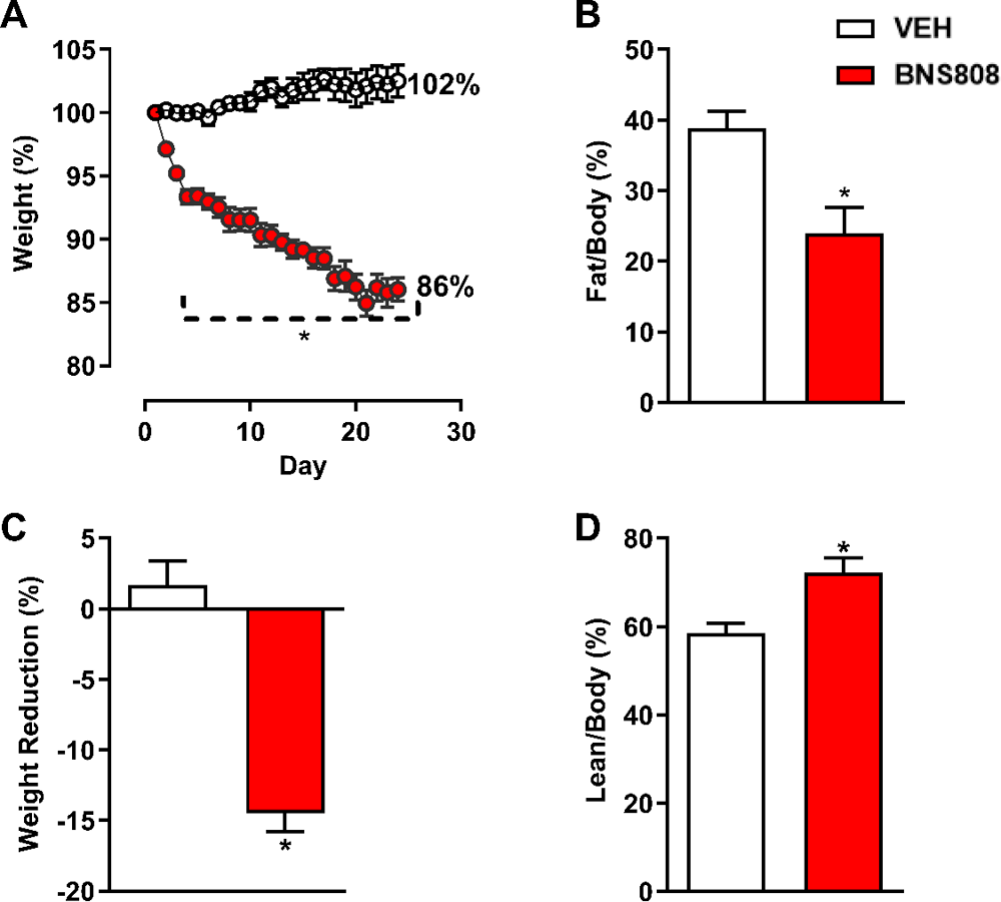
Peripheral CB_1_R blocker, BNS808, is effective in reducing obesity. BNS808 (1 mg/kg/day for 24 days) reduced body weight (A, B) and fat mass (C) and increased lean body mass (D) in diet-induced obese mice. Data represent the mean ± SEM from 6 mice per group. *P < 0.05 vs vehicle-treated control.

**Figure 4:**
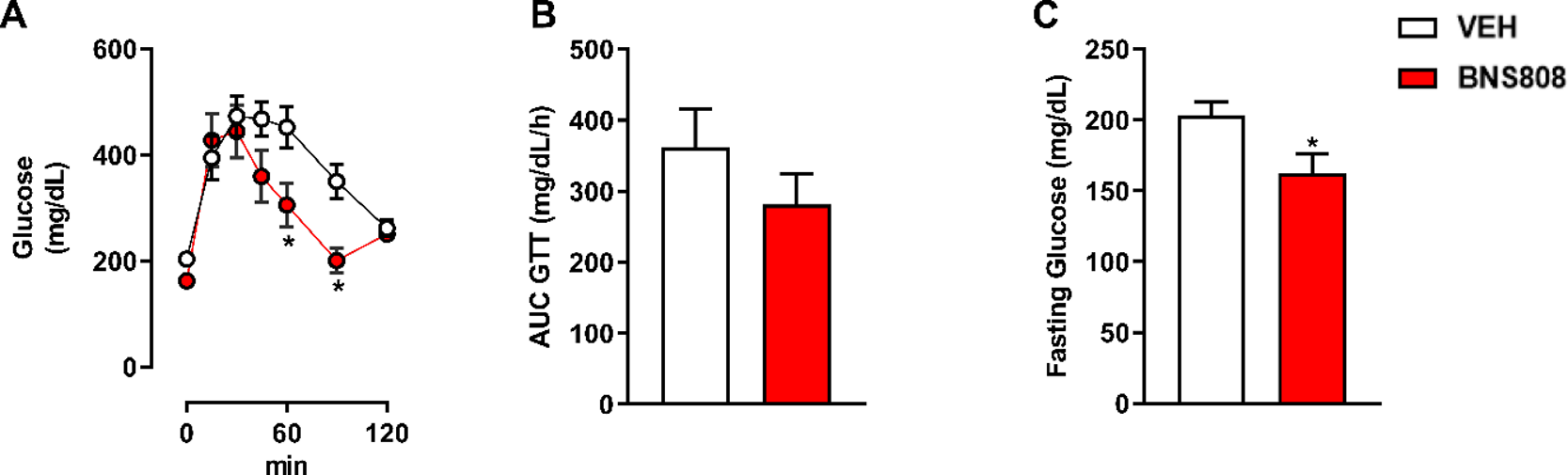
Peripheral CB_1_R blocker, BNS808, affects glucose hemostasis. BNS808 (1 mg/kg/day for 24 days) showed a trend in reducing glucose intolerance (A, B) and decreased fasting blood glucose levels (C). Data represent the mean ± SEM from 6 mice per group. *P < 0.05 vs vehicle-treated control.

Next, the potential of BNS808 to affect whole-body systemic metabolism was assessed in diet-induced obese (DIO) mice. Male C57Bl/6J mice were kept on a diet containing 60% of calories as fat, leading to obesity (body weight >42 g) after 16 weeks. DIO mice were then chronically treated with 1 mg/kg/day of BNS808 oral doses for 24 days. The results showed that BNS808 treatment reduced body weight compared to the control group (Figure 3A,B). This decrease in body weight was attributed to a reduction in total body fat, with a significant increase in lean body mass, as determined by EchoMRI analysis (Figure 3C,D). In addition, there was a trend toward substantial effects of the drug on glucose homeostasis (Figure 4A,B). Notably, the drug reduced the glucose levels in fasting conditions (Figure 4C).

**Figure 5:**
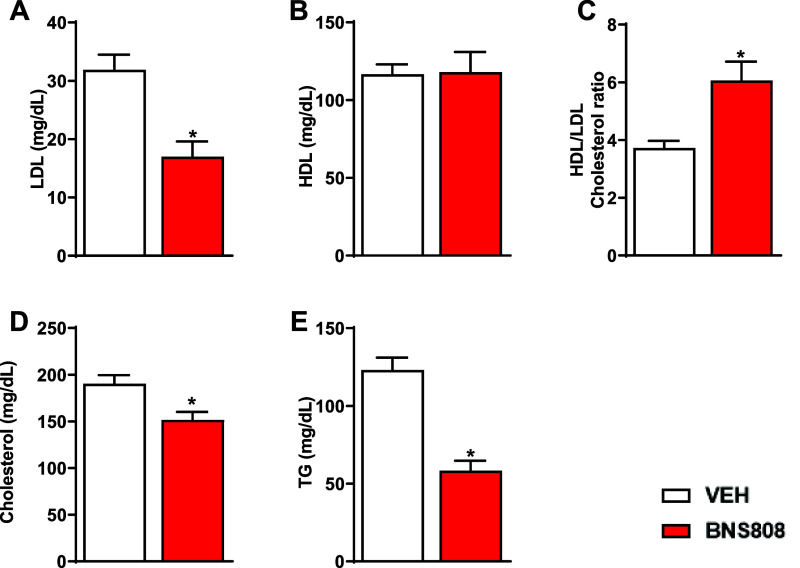
Peripheral CB_1_R blocker, BNS808, improves dyslipidemia. BNS808 (1 mg/kg/day for 24 days) reduced LDL levels (A) without affecting HDL levels (B), resulting in increased HDL/LDL ratio (C). BNS808 also reduced total cholesterol (D) and triglycerides (E) levels. Data represents the mean ± SEM from 6 mice per group. *P < 0.05 vs vehicle-treated control.

**Figure 6:**
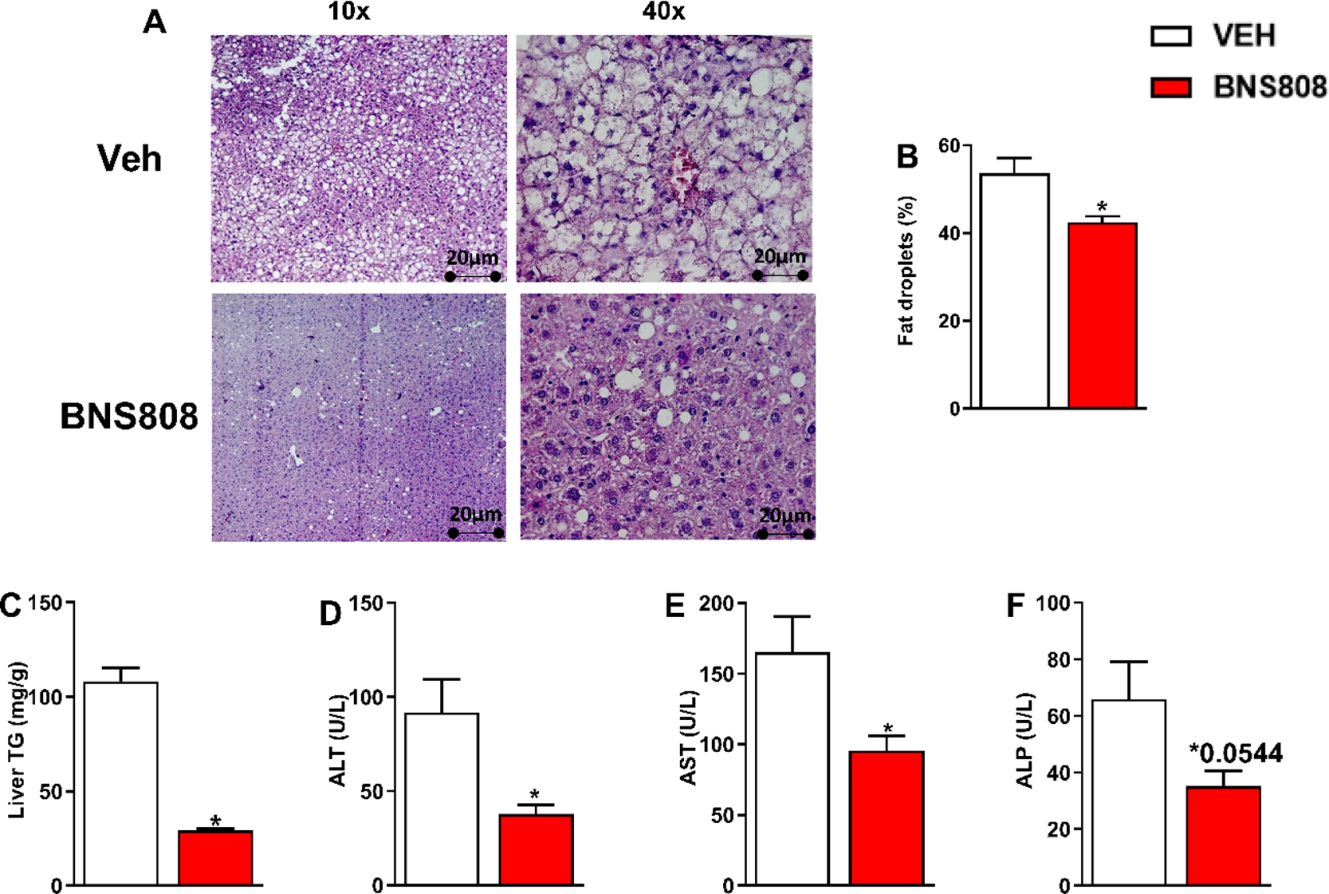
Chronic BNS808 administration (1 mg/kg/day for 24 days) reduces HFD-induced hepatic steatosis and liver injury in diet-induced obese mice. An elevated fat vacuole deposition, measured in H&E sections (A), was evident in the DIO mice treated with vehicle compared with the BNS808-treated animals on the same diet (B). Furthermore, a decrease in triglyceride content in the liver (C) as well as reductions in liver enzyme levels (ALT, AST, and ALP), measured by the COBAS C-111 Chemistry analyzer, were noticeable in the BNS808-treated mice (D–F). Data represent the mean ± SEM from at least 5 mice per group. *P < 0.05 vs vehicle-treated control.

Our findings also indicate that hypertriglyceridemia and hypercholesterolemia were ameliorated by BNS808 (Figure 5A–E). Interestingly, BNS808 demonstrated efficacy in reversing hepatic steatosis and hepatocellular damage, as evidenced by histological assessment (Figure 6A). Furthermore, treatment with BNS808 led to a significant reduction in liver triglyceride content (Figure 6B) and liver enzymes, including ALT, AST, and ALP (Figure 6C–E). These findings suggest that BNS808 shows promise in improving the metabolic profile in DIO mice.

## Discussion and Conclusions

CB_1_R antagonism has emerged as a promising therapeutic approach for various conditions, including diabetes, obesity, fatty liver, and dyslipidemias.^39,58^ However, their undesirable adverse effects on the CNS hinder the clinical utility of non-selective, globally acting CB1R antagonists.^31,59,60^ Since CB_1_Rs are also present in peripheral organs,^61^ targeting these receptors in the periphery has shown potential in experimental models of these diseases. Consequently, ongoing research aims to develop peripherally restricted CB_1_R antagonists for therapeutic applications, with some compounds that are currently under clinical assessment. While progress has been made in this field, further refinement and validation of these presently disclosed peripherally restricted CB_1_R blockers are needed before promoting them toward clinical use.

In this study, we developed novel peripheral-selective CB_1_R antagonists that could overcome the limitations of centrally acting CB_1_R blockers. Specifically, we describe synthesizing and characterizing a series of BNS compounds designed as peripherally selective CB_1_R blockers based on the 10q (BNS8) scaffold (compound 8).^56^ We focused on incorporating functional groups such as benzenesulfonamide and carboxyamide, which have shown promise in improving CB_1_R selectivity and affinity. The synthesis of novel CB_1_R blockers involved key transformations such as nucleophilic substitutions, sulfonylation, and amidation. Mechanistic insights into the bond-forming steps illustrate the impact of these modifications on receptor binding. The sulfonamide and carboxyamide functionalizations were designed to enhance selectivity by improving molecular interactions with CB_1_R while minimizing interactions with CB_2_R affinity.^62^ The synthesized BNS compounds were fully characterized using different analytical techniques, including NMR spectroscopy, mass spectrometry, and HPLC. NMR spectroscopy confirmed the compounds’ structure and assessed their purity (Supplementary Figures 3–8). The results indicated that the final BNS compounds were obtained in good yields and with high purity, indicating the effectiveness of the synthetic route employed in this study. The selection of sulfonyl chlorides, amines, and specific base catalysts focused on their capacity to optimize reaction yields and improve selectivity. The impact of electronic substituents on reactivity and the stability of the final compound was thoroughly assessed, using mild reaction conditions to prevent side reactions and ensure high purity.^63^

Our research has successfully evaluated the physiochemical parameters and conditions necessary to develop highly peripheral selective CB_1_R antagonists. Key factors such as lipophilicity, *M*_W_, functional groups, substrate mode, stereochemistry, topological polar surface area (TPSA), HBD, ionization mode, and structure–activity relationships (SAR) were carefully considered to ensure peripheral selectivity. Neglecting some of these factors could compromise the development of effective therapeutic agents. Our multi-pronged strategy involved selecting building blocks that generate charged compounds with high TPSAs, leading to the discovery of CB_1_R antagonists with strong selectivity for CB_1_R over CB_2_R. By choosing the appropriate building blocks and conjugated groups, we synthesized CB_1_R antagonists with a *M*_W_ over 500 and a lipophilicity above 5.5, ensuring the new molecules are directed toward peripheral CB_1_Rs. While our compounds have HBD values of 1 or 2, which may seem inconsistent with the desired properties, these values were balanced by other parameters, including *M*_W_, clogP, and TPSA, contributing to peripheral selectivity. The high lipophilicity of compounds such as BNS808 does not preclude peripheral activity; instead, it reflects an intentional optimization of the SAR. This design incorporates specific functional groups and a unique chemical scaffold to enhance receptor binding affinity while limiting BBB penetration. Specifically, the sulfonamide group is a critical pharmacophoric element in CB_1_R antagonists, contributing to both receptor binding and drug-like properties. As a hydrogen bond donor and acceptor, it enhances ligand interactions with key CB_1_R residues, improving binding affinity and selectivity.^64−66^ Additionally, its electron-withdrawing nature strengthens ligand–receptor interactions, while its high polarity optimizes solubility and bioavailability. Importantly, sulfonamide modifications can modulate lipophilicity and TPSA, reducing blood–brain barrier penetration and enhancing peripheral selectivity.^67−70^ Indeed, in our study, this group plays a key role in fine-tuning pharmacokinetics and pharmacodynamics, offering opportunities to improve metabolic stability and therapeutic potential. Therefore, by leveraging a combination of parameters beyond hydrophobicity, BNS808 demonstrates the feasibility of achieving peripheral selectivity without strictly adhering to conventional molecular design paradigms.

Our study also assessed the potential of BNS compounds to serve as substrates of P-gp, which plays a crucial role as a gatekeeper in the brain,^71^ actively preventing the entry of foreign substances into the brain and facilitating their efflux into the bloodstream. Our results revealed that BNS808 and the reference parent compound 10q (BNS8) demonstrated characteristics consistent with P-gp substrates. This attribute is desirable for peripherally restricted CB_1_R blockers, suggesting that these compounds are less likely to penetrate the BBB and exert CNS side effects. However, it is worth noting that carboxyamide moiety in the compounds, such as observed in BNS816, may influence their P-gp substrate status. This observation suggests that specific structural features of the BNS compounds can affect their interaction with P-gp and subsequent permeability across the BBB.

Our study revealed the discovery of several potent and highly selective CB_1_R antagonists within the BNS compound series. Notably, these compounds exhibited *K*_i_ (or IC_50_) values below 100 nM and demonstrated selectivity exceeding 100-fold compared to CB_2_R. The potency at CB_1_R was achieved by incorporating various functional groups, including benzenesulfonamide and carboxyamide, highlighting the versatility of the synthetic approach. Among these compounds, BNS808 emerged as promising, demonstrating high binding affinity and remarkable selectivity for CB_1_R, positioning it as a leading candidate for further development.

Given the prominent involvement of the liver in the pathogenesis of obesity and its metabolic complications,^5,72^ it was imperative to assess the potential hepatotoxicity of compounds before testing their metabolic efficacy in reversing MASLD. In response to this necessity, we conducted cell viability and mini-Ames assays to elucidate the impact of the compounds on hepatic cells. Our results reveal that the compounds, particularly BNS808, require elevated concentration to elicit discernible hepatic toxicity. This observation underscores the compounds’ favorable safety profiles, especially concerning hepatic effects. As per the cardiotoxicity aspect, particularly QT prolongation, which is critical in the drug development process,^73^ we next conducted an hERG potassium channel assay to evaluate the cardiotoxic potential of BNS808. Our findings indicated a low probability of cardiotoxicity associated with this compound. Interestingly, studies have suggested that pharmacological inhibition of the CB_1_R may offer protection against doxorubicin-induced cardiotoxicity,^74^ and the CB_1_R antagonist rimonabant has been shown to protect against acute myocardial infarction.^75^

As noted earlier, BNS808 is a P-gp substrate, which may affect its bioavailability.^76^ Nevertheless, we propose that appropriate drug formulation can overcome this challenge and enhance the compound’s bioavailability. Through our efforts, we have successfully developed a unique formulation to improve the pharmacokinetic (PK) parameters of BNS808. Subsequent assessment of the PK properties of BNS808, including efficacy and distribution, was conducted in vivo. Our finding revealed that BNS808 exhibited peripheral restriction, indicating its inability to penetrate the BBB and thus mitigate the adverse effects commonly associated with centrally acting CB_1_R antagonists. The biodistribution studies involved the acute administration of BNS808 to mice, and then measuring its concentration in various tissues showed that it has an excellent distribution in peripheral tissues like the liver with minimal exposure in the brain, indicating its potential as a peripherally selective CB_1_R blocker.

Drug–drug interactions, often linked to the inhibition or induction of cytochrome P450 (CYP) enzymes, are a significant concern in drug development.^77^ To address this, we conducted in vitro CYP inhibition assays using liver microsomes. Unlike rimonabant, which is metabolized through CYP3A and amidohydrolase pathways and shows sensitivity to CYP3A modulators,^78^ BNS808 exhibits moderate inhibition of CYP3A4 and weak to moderate inhibition of CYP2C9 and CYP2C19. These findings suggest that BNS808 has a reduced likelihood of causing significant drug–drug interactions compared to rimonabant.

Our study demonstrated that the peripherally restricted CB_1_R antagonist, BNS808, effectively mitigated body weight and adiposity in DIO mice. These results strongly suggest that the observed effects are mediated primarily through the blockage of peripheral CB_1_R rather than central action. This supports previous indications that the hyperphagic and weight-reducing effects of brain-penetrant CB_1_R inverse agonists may, in part, be attributed to the blockade of peripheral CB_1_R.^16,79^ Furthermore, our research demonstrates that BNS808 treatment normalizes liver enzyme levels in DIO mice, indicating improved liver function. Additionally, we observed a significant reduction in liver fat content and decreased triglyceride levels in the liver. These findings are consistent with previous studies highlighting the liver function-improving properties of CB_1_R blockers.^14,80^ It is plausible that in obesity, the fraction of CB_1_Rs in the active conformation is elevated in peripheral tissues involved in metabolic regulation.^79,81,82^ This heightened activity may explain the efficacy of peripherally restricted CB_1_R blockers.

Incorporating benzenesulfonamide, known for its carbonic anhydrase (CA) inhibitory activity,^83^ into the structure of BNS808 presents a compelling avenue for potential therapeutic augmentation, as highlighted by previous research.^84,85^ CAs, traditionally recognized for their role in pH regulation and buffering across various cellular and tissue contexts, have progressively emerged as significant players in metabolic pathways.^86^ This expanded role in metabolic processes, observed in neoplastic conditions.^87,88^ Typical cellular environments encompass critical functions such as fatty acid biosynthesis and de novo lipogenesis (DNL).^89^ Notably, these metabolic processes involve events spanning mitochondrial and cytosolic domains, implicating several enzymes within the Krebs cycle and DNL pathways. Among these, pyruvate carboxylase (PC) and acetyl-coenzyme A carboxylase (ACC) are notable for utilizing bicarbonate as a substrate, distinct from the conventional CO_2_.^84^ Seminal research from the 1990s firmly established that perturbation of mitochondrial and cytosolic CAs could disrupt fatty acid biosynthesis and DNL across a spectrum of cellular systems, tissues, and animal models.^90−92^ Moreover, it is pertinent to consider that BNS808 may interact with additional molecular targets beyond its primary engagement with CB_1_R, particularly CAs. A comprehensive exploration of these interactions is imperative to elucidate the primary drivers underlying the metabolic effects of BNS808, whether primarily attributable to CB_1_R blockade, CA inhibition, or a synergistic interplay of both mechanisms. Further investigations in this realm are thus warranted to unravel the intricate molecular underpinnings of BNS808’s therapeutic potential.

Our findings demonstrate that rationally designing and engineering chemical-physical properties from the outset enabled the successful synthesis and characterization of a series of BNS compounds selectively targeting peripheral CB_1_R. BNS808 exhibits high binding affinity and selectivity for peripheral CB_1_R and favorable pharmacokinetic, safety, and pharmacological profiles. This compound library provides a promising foundation for identifying additional peripherally selective CB_1_R antagonists with therapeutic potential. Furthermore, the data strongly support the potential of BNS808 as a therapeutic agent for treating obesity and its associated metabolic disorders. Its peripherally selective nature may significantly reduce the risk of adverse effects typically linked to centrally acting CB_1_R antagonists. Nonetheless, further studies are needed to elucidate the mechanisms underlying BNS808’s metabolic effects and to optimize its therapeutic efficacy.

## Experimental Section

### General Procedures

All compounds were synthesized and characterized using HPLC, NMR spectroscopy, and mass spectrometry. Purity >95% was confirmed via reverse-phase HPLC, and reaction monitoring was performed using TLC. The clogP values of the compounds were calculated using ChemDraw. Before synthesizing the BNS8 analogs, we initiated the process by synthesizing Building Block 8 (BB8): (*R*,*S*)-2-(2-chlorophenyl)-3-(4-chlorophenyl)-5,6,7,8-tetrahydro-2*H*-oxepino[3,2-*c*]pyrazol-8-aminium chloride.^56^ The hydrochloric acid salt of BNS8 was then prepared following established protocols.

### Synthesis and Characterization of BNS8 Analogs

Two general procedures were employed to synthesize seven different molecules (Scheme 3), the diverse properties of which are detailed in Tables 1 and 2.

### General Procedure A, as Depicted in Scheme 1

Procedure A, was chosen to synthesize the analogs BNS807, BNS808, BNS809, and BNS813. The reaction commenced by dissolving (*R*,*S*)-2-(2-chlorophenyl)-3-(4-chlorophenyl)-5,6,7,8-tetrahydro-2*H*-oxepino[3,2-*c*]pyrazol-8-aminium chloride (BNS8) in dichloromethane (DCM) at a concentration of 0.1 M. The solution was then cooled in an ice bath, and the appropriate sulfonyl chloride derivatives (at 1.2 equiv) and triethylamine (TEA; also, at 1.2 equiv) were added. The progression of the reaction was monitored via thin-layer chromatography (TLC) using a mobile phase of 2% methanol in DCM until completion. Subsequently, the mixture was diluted with additional DCM (50–100 mL) and subjected to washing with a saturated sodium bicarbonate solution and brine. The organic layer was desiccated over anhydrous sodium sulfate, filtered, and concentrated to obtain the crude product. Depending on the synthesized compound, the crude product was purified through flash chromatography utilizing silica or reversed phase (RP) silica. Final chemical properties, including compound purity, were determined and characterized using HPLC-MS and ^1^H NMR.

### General Procedure B, as Depicted in Scheme 2

Procedure B, was chosen to synthesize the analogs BNS815, BNS816, and BNS825. The synthesis of Intermediate-1 (BNS8-INT-1), *N*-(2-(2-chlorophenyl)-3-(4-chlorophenyl-5,6,7,8-tetrahydro-2*H*-oxepino[3,2-*c*]pyrazol-8-yl)piperidine-4-carboxamide, was carried out according to procedure B illustrated in Scheme 2. Piperidine-2-carboxylic acid was dissolved in DCM. Triethylamine (TEA) and hydroxybenzotriazole (HOBt) were added at equimolar concentrations. The solution was cooled in an ice bath, and equimolar amounts of *N*-(3-(Dimethylamino)propyl)-*N*′-ethyl carbodiimide hydrochloride (EDC) was added for activation. Subsequently, the building block, (*R*,*S*)-2-(2-chlorophenyl)-3-(4-chlorophenyl)-5,6,7,8-tetrahydro-2*H*-oxepino[3,2-*c*]pyrazol-8-aminium chloride (BNS8), at equimolar concentration, was introduced. The reaction was stirred overnight. The progress of the reaction was monitored by TLC (2% methanol in DCM) to ensure completion. Following completion, the reaction mixture was diluted with additional DCM (50–100 mL) and subjected to washing with a saturated sodium bicarbonate solution and brine. The organic layer was then desiccated over anhydrous sodium sulfate, filtered, and concentrated to yield the crude product. The product was purified by flash chromatography on either silica or RP silica, as indicated for each compound. Final chemical properties, including compound purity, were analyzed and characterized by HPLC-MS and ^1^H NMR.

### Synthesis of BNS807

(*S*)-*N*-(2-(2-Chlorophenyl)-3-(4-chlorophenyl)-4,5,6,7,8,9-hexahydro-2*H*-cycloocta[*c*]pyrazol-9-yl)-4-methylbenzene sulfonamide was synthesized following the general procedure A, starting with BNS8 (150 mg, 0.37 mmol), tosyl chloride (104 mg, 0.55 mmol), and triethylamine (TEA; 153 μL, 1.1 mmol). The reaction yielded the desired product, purified using RP C18 flash purification. This process resulted in the isolation of 190 mg of BNS807, yielding 98% and purity of 98%. The ^1^H NMR spectorum (500 MHz, DMSO-d_6_) revealed characteristic signals at δ 1.75–1.93 (m, 3H), 2.19 (dt, *J* = 17.6, 10.0 Hz, 1H), 2.31 (s, 3H), 3.83–3.90 (m, 1H), 4.03 (dd, *J* = 11.0, 6.7 Hz, 1H), 4.53 (s, 1H), 7.06 (d, *J* = 8.6 Hz, 2H), 7.27 (d, *J* = 8.0 Hz, 2H), 7.32–7.40 (m, 3H), 7.45–7.54 (m, 3H), 7.67–7.72 (m, 2H), 8.09 (d, *J* = 7.0 Hz, 1H).

### Synthesis of BNS808

The compound 4-chloro-*N*-[2-(2-chlorophenyl)-3-(4-chlorophenyl)-5,6,7,8-tetrahydrooxepino[3,2-*c*]pyrazol-8-yl]benzenesulfonamide was synthesized using general procedure A, starting with building block BNS8 (150 mg, 0.37 mmol), 4-chlorobenzenesulfonyl chloride (116 mg, 0.55 mmol), and TEA (153 μL, 1.1 mmol). The reaction yielded the desired product, which was then purified using RP C18 flash purification, resulting in 190 mg of BNS808 with a 98% yield and purity of 99%. The ^1^H NMR spectrum (500 MHz, Chloroform-d) showed signals at δ 1.87 (dtd, *J* = 13.4, 10.8, 2.5 Hz, 1H), 1.97–2.06 (m, 1H), 2.09–2.17 (m, 1H), 2.39–2.48 (m, 1H), 3.72 (dd, *J* = 11.8, 9.8 Hz, 1H), 4.22 (ddd, *J* = 12.0, 5.6, 2.5 Hz, 1H), 4.38 (ddd, *J* = 10.5, 5.7, 3.5 Hz, 1H), 5.95 (d, *J* = 5.6 Hz, 1H), 7.01–7.07 (m, 2H), 7.18–7.24 (m, 3H), 7.33–7.41 (m, 4H), 7.44 (dd, *J* = 7.8, 1.8 Hz, 1H), 7.82–7.87 (m, 2H).

### Synthesis of BNS809

*N*-[2-(2-Chlorophenyl)-3-(4-chlorophenyl)-5,6,7,8-tetrahydrooxepino[3,2-*c*]pyrazol-8-yl]methanesulfonamide was synthesized using the general procedure A. The reaction involved BNS8 (150 mg, 0.37 mmol), mesyl chloride (42 μL, 0.55 mmol), and TEA (153 μL, 1.1 mmol). The resulting product was purified through RP C18 flash purification, yielding 160 mg of BNS809 with a yield of 97% and purity of 97%. The ^1^H NMR spectrum (500 MHz, Chloroform-d) exhibited signals at δ 1.96 (q, *J* = 11.1 Hz, 1H), 2.05–2.14 (m, 1H), 2.18 (dt, *J* = 14.3, 6.9 Hz, 1H), 3.01 (s, 3H), 3.87 (t, *J* = 10.5 Hz, 1H), 4.18 (q, *J* = 7.8, 6.7 Hz, 1H), 4.71 (ddd, *J* = 10.0, 7.1, 3.4 Hz, 1H), 5.36 (d, *J* = 7.2 Hz, 1H), 7.09–7.13 (m, 2H), 7.22–7.25 (m, 2H), 7.32–7.42 (m, 3H), 7.42–7.46 (m, 1H).

### Synthesis of BNS813

1-[2-(2-Chlorophenyl)-3-(4-chlorophenyl)-5,6,7,8-tetrahydrooxepino[3,2-*c*]pyrazol-8-yl]-3-methyl-urea was synthesized following the general procedure A. The reaction involved BNS8 (150 mg, 0.37 mmol), methyl-carbamic chloride (51 mg, 0.55 mmol), and TEA (153 μL, 1.1 mmol). The resulting product was then purified using silica flash purification, resulting in 156 mg of BNS813 with a yield of 99% and purity of 97%. The ^1^H NMR spectrum (500 MHz, Chloroform-d) exhibited signals at δ 1.75 (dq, *J* = 13.7, 7.5, 6.9 Hz, 1H), 2.09–2.18 (m, 2H), 2.29–2.38 (m, 1H), 2.76 (s, 3H), 3.80–3.90 (m, 1H), 4.18 (dt, *J* = 11.9, 4.4 Hz, 1H), 4.93 (dd, *J* = 9.4, 3.2 Hz, 1H), 5.67 (s, 1H), 7.10–7.14 (m, 2H), 7.21–7.24 (m, 2H), 7.30–7.39 (m, 3H), 7.43 (dt, *J* = 7.0, 1.5 Hz, 1H).

### Synthesis of BNS815

*N*-[2-(2-Chlorophenyl)-3-(4-chlorophenyl)-5,6,7,8-tetrahydrooxepino[3,2-*c*]pyrazol-8-yl]-1-methylsulfonyl-piperidine-4-carboxamide was synthesized using the general procedure B. The reaction involved the use of BNS8-INT-1 (90 mg, 0.19 mmol) (which was synthesized by procedure B, Scheme 2), mesyl chloride (22 μL, 0.28 mmol), and TEA (39 μL, 0.28 mmol). The product was subsequently purified using silica flash purification, resulting in 94 mg of BNS815 with a yield of 90% and purity of 99%. The ^1^H NMR spectrum (500 MHz, DMSO-d_6_) exhibited signals at δ 1.55–1.66 (m, 2H), 1.78 (dd, *J* = 27.6, 12.7 Hz, 2H), 1.84–1.96 (m, 3H), 2.06–2.15 (m, 1H), 2.38 (ddd, *J* = 13.8, 8.8, 3.1 Hz, 1H), 2.68 (dt, *J* = 12.0, 8.7 Hz, 2H), 3.54 (t, *J* = 11.3 Hz, 2H), 3.94 (t, *J* = 9.9 Hz, 1H), 4.09 (dd, J = 11.7, 6.5 Hz, 1H), 5.08 (dt, *J* = 11.7, 4.3 Hz, 1H), 7.14 (d, *J* = 8.6 Hz, 2H), 7.39 (d, *J* = 8.5 Hz, 2H), 7.45–7.52 (m, 2H), 7.55 (dq, *J* = 7.1, 3.8 Hz, 2H), 8.30 (d, *J* = 8.1 Hz, 1H).

### Synthesis of BNS816

Ethyl-4-((2-(2-chlorophenyl)-3-(4-chlorophenyl)-5,6,7,8-tetrahydro-2*H*-oxepino[3,2-*c*]pyrazol-8-yl)carbamoyl)piperidine-1-carboxylate was synthesized following the general procedure B. The reaction involved BNS8-INT-1 (90 mg, 0.19 mmol), (which was synthesized by procedure B, Scheme 2) ethyl chloroformate (26 μL, 0.28 mmol), and TEA (39 μL, 0.28 mmol). The resulting product was purified using silica flash purification, resulting in 98 mg of BNS816 with a yield of 95% and purity of 98%. The ^1^H NMR spectrum (500 MHz, DMSO-d_6_) exhibited signals at δ 1.17 (t, *J* = 7.1 Hz, 3H), 1.36–1.48 (m, 2H), 1.66 (dd, *J* = 28.2, 12.6 Hz, 2H), 1.81–1.97 (m, 3H), 2.10 (s, 1H), 2.45 (ddt, *J* = 11.4, 7.5, 3.7 Hz, 1H), 2.76 (s, 2H), 3.89–3.99 (m, 3H), 4.02 (q, *J* = 7.1 Hz, 2H), 4.07 (dd, *J* = 11.6, 6.6 Hz, 1H), 5.07 (td, *J* = 7.8, 3.9 Hz, 1H), 7.14 (d, *J* = 8.6 Hz, 2H), 7.39 (d, *J* = 8.6 Hz, 2H), 7.45–7.52 (m, 2H), 7.52–7.58 (m, 2H), 8.25 (d, *J* = 8.0 Hz, 1H).

### Synthesis of BNS825

*N*-[2-(2-Chlorophenyl)-3-(4-chlorophenyl)-5,6,7,8-tetrahydrooxepino[3,2-*c*]pyrazol-8-yl]-1-(4-chlorophenyl)sulfonyl-piperidine-4-carboxamide was synthesized following the general procedure B. The reaction involved the use of BNS8-INT-1 (85 mg, 0.18 mmol) (which was synthesized by procedure B, Scheme 2), 4-chlorobenzenesulfonyl chloride (55 mg, 0.26 mmol), and TEA (49 μL, 0.35 mmol). The resulting product was purified using RP C18 flash purification, producing 91 mg of BNS825 with a yield of 79% and purity of 100%. The ^1^H NMR spectrum (500 MHz, Chloroform-d) exhibited signals at δ 1.61 (q, *J* = 12.2, 11.6 Hz, 1H), 1.82–1.99 (m, 4H), 2.01–2.10 (m, 1H), 2.10–2.18 (m, 1H), 2.18–2.25 (m, 1H), 2.36–2.49 (m, 3H), 3.64–3.81 (m, 3H), 4.25–4.33 (m, 1H), 5.09 (ddd, *J* = 10.2, 6.3, 3.2 Hz, 1H), 6.87 (bs, 1H), 7.12 (d, *J* = 8.6 Hz, 2H), 7.24 (d, *J* = 8.6 Hz, 2H), 7.32–7.45 (m, 4H), 7.49 (d, *J* = 8.6 Hz, 2H), 7.68 (d, *J* = 8.6 Hz, 2H).

### BNS808’s Solubility and Stability in Various Aqueous Media

To prepare simulated gastric fluid (SGF), 500 mg of NaCl and 800 mg of pepsin were accurately weighed and transferred to a 250 mL volumetric flask. Subsequently, 1.75 mL of concentrated HCl (37%) was added to dissolve the compound. The solution was then diluted with water to reach a final volume of 250 mL. The pH of the SGF was measured using a pH meter, and the target pH was adjusted to 1.199.

To prepare simulated intestinal fluid (SIF), 1.7 g of KH_2_PO_4_ was weighed and dissolved in 62.5 mL of water. Then, 19.25 mL of 0.2 N NaOH and 125 mL of water were added to the solution. Subsequently, 2.5 g of pancreatin was mixed into the solution. The pH of the solution was adjusted to 6.8 using either 0.2 N NaOH or 0.2 N HCl. Finally, the volume was adjusted to 250 mL.

The test was conducted using 250 mL SGF with a pH of 1.2 or SIF, employing a paddle rotating at 75 rpm and maintaining a bath temperature of 37 °C. After 2 h in the SGF, each sample was retrieved, and the SGF was also collected simultaneously. A 5 mL sample was extracted from the clay vessel using a glass syringe and filtered through a nylon filter (0.45 μm, 25 mm) into labeled glass tubes. The solution underwent analysis via HPLC, and the release percentage (%) was determined using the following equation: Release (%) = (Released amount/Labeled amount) × 100.

### Radioligand Binding Assays

BNS compounds’ binding to CB_1_R and CB_2_R was assessed in competition displacement assays using [^3^H]CP-55,940 as the radioligand and crude membranes from mouse brain or human cells overexpressing the CB_1_R or CB_2_R. All data were in triplicates with *K*_i_ values determined from three independent experiments. See SI for a detailed procedure.

### [^35^S]GTPγS Binding

Mouse brains were dissected, and P2 membranes were prepared and resuspended at ∼10 μg protein/μL in 350 μL assay buffer containing 0.14% BSA. Ligand-stimulated [^35^S]GTPγS binding was assayed as described in the SI.

### Bidirectional Permeability Assessment in MDR1-MDCK Cells

MDR1-MDCKII cells were seeded onto polyethylene membranes (PET) in a 96-well insert system. Test and reference compounds were administered to the apical (A-to-B) and basolateral (B-to-A) sides of the cell monolayer. Permeation of the compounds in both directions was assessed, with and without a P-glycoprotein (P-gp) inhibitor (GF120918). Nadolol and metoprolol were employed as low and high permeability markers, respectively, while digoxin served as a reference compound for a P-gp substrate evaluation. After a 2.5-h incubation period, each compound’s permeability and efflux ratio were calculated as described in the SI.

### CYP Inhibition

Human liver microsomes were incubated with the test compounds at 0.2 mg/mL of microsomal protein. The incubation was conducted at 37 °C in the presence of a NADPH (β-Nicotinamide adenine dinucleotide phosphate reduced form) regenerating system at a concentration of approximately 10 mM. To assess CYP inhibition, positive control substrates were utilized, including Phenacetin (1A2 substrate), Diclofenac (2C9 substrate), S-mephenytoin (2C19 substrate), Dextromethorphan (2D6 substrate), and Midazolam (3A4 substrate). The mixture was centrifuged after a 10 min incubation period to precipitate the protein. Subsequently, the samples underwent LC-MS/MS analysis, and the IC50 values were calculated to ascertain the inhibitory potency of the test compounds on specific CYP enzymes.

### Animal and Experimental Procedure

The experimental procedures were ethically approved by the Institutional Animal Care and Use Committee of the Hebrew University, an AAALAC International accredited institute (Ethic Approval #MD-22-17031-3). Male 6-week-old C57Bl/6J mice were procured from Harlan, Israel, and were housed under standard conditions with a 12-h light/dark cycle, with *ad libitum* access to food and water.

To induce diet-induced obesity, the mice were fed a high-fat diet (HFD) containing 60% of calories from fat, 20% from protein, and 20% carbohydrates (Research Diet, D12492) for 16 weeks. HFD-fed obese mice were administered either vehicle or BNS808 (in SEDDs) orally at 1 mg/kg (dosing volume of 1 mL/kg) for 24 days. Daily monitoring of body weight was conducted throughout the experimental period. Total body fat and lean masses were assessed using the EchoMRI-100H (Echo Medical Systems LLC, Houston, TX, USA). At the end of week 20, mice were euthanized via cervical dislocation under anesthesia. Various tissues, including the kidneys, brain, liver, fat pads, and muscles, were harvested and snap-frozen or fixed in buffered 4% formalin. Trunk blood samples were collected for biochemical parameters analysis.

### Self-Emulsifying Drug Delivery System (SEDDs)

SEDDs were prepared using a mixture of caproyl 90 (25%), Cremophor EL (25.73%), propylene glycol (12.87%), MCT (28.51%), and ethanol (7.89%). Then, an adequate amount of BNS808 was dissolved in the SEDDs base to achieve a BNS808 concentration of 0.1%.

### Glucose Tolerance (ipGTT) Test

Mice subjected to an overnight fast were administered glucose (1.5 g/kg, IP), after which tail blood samples were collected at 0, 15, 30, 45, 60, 90, and 120 min post-injection. Blood glucose concentrations were measured using the Elite glucometer (Bayer, Pittsburgh, PA), allowing for the assessment of glucose tolerance over the specified time intervals.

### Blood and Liver Biochemistry

Serum levels of alanine transaminase (ALT), aspartate transaminase (AST), alkaline phosphate (ALP), high-density lipoprotein (HDL), and low-density lipoprotein (LDL) were determined using the Cobas C-111 chemistry analyzer (Roche, Switzerland). Upon animal sacrifice, liver tissue was extracted according to the previously established protocol.^79^ The extracted liver tissue was subsequently assessed for triglyceride contents using the same chemistry analyzer.

### Histopathological Analyses

Paraffin-embedded liver sections, 5 μm thick, were prepared from 5 animals per group and subjected to hematoxylin-eosin (H&E) staining. This was carried out on a separate batch of animals of the same age as those used for other assays. These animals underwent the same procedures and treatments outlined in the animal and experimental method. Liver images were captured with a Zeiss AxioCam ICc5 color camera attached to a Zeiss Axio Scope.A1 light microscope. Images were captured from 10 random 40× magnifications for each animal. Analysis of the fat droplet in the H&E staining was done by ImageJ according to the following macro:

run(“RGB Stack”); setSlice(2);

run(“Set Scale···″, “distance = 588.0136 known = 200 unit = um”);

setAutoThreshold(“Default dark no-reset”);

getThreshold(min, max)

setThreshold(max/1.75, max);

run(“Measure″);

### Multi-parameter Activity Assessment

The activity profiles of the mice were evaluated using the Promethion High-Definition Behavioral Phenotyping System (Sable Instruments, Inc., Las Vegas, NV, USA). MetaScreen software version 2.2.18.0 was employed for data acquisition and instrument control, while ExpeData version 1.8.4 was used for processing raw data. An analysis script detailing all aspects of the data transformation was utilized. Mice with unrestricted access to food and water were subjected to a standard 12 h light/12 h dark cycle, comprising a 16-h acclimation period followed by 24 h of sampling. Ambulatory activity and position were concurrently quantified by the number of disruptions of the infrared beams in three dimensions (*XYZ*) with a beam spacing of 0.25 cm.

#### Assessment of Hyperactivity

This experiment aimed to evaluate the capacity of BNS808 and rimonabant (used as a positive control) to elicit centrally mediated hyperactivity in mice (a known feature of a brain penetrant compound). For this purpose, male wild-type C57BL/6J mice received a single dose of either the vehicle, rimonabant (10 mg/kg, PO), or BNS808 (1 or 10 mg/kg, PO). The locomotor activity was quantified by measuring the disruptions of infrared beams in two dimensions (*XY*) for 1 h. Each testing session involved evaluating two groups at a time.

#### Evaluation of CB_1_R-Induced Hypomotility

The objective of this experiment was to evaluate the capability of BNS808 and rimonabant (utilized as a positive control) in inhibiting hypomotility induced by a CB_1_R agonist (HU210). Male wild-type C57BL/6J mice were administered a single dose of either the vehicle, rimonabant (10 mg/kg, PO), or BNS808 (1 or 10 mg/kg, PO). Thirty minutes following this administration, the mice received a single dose of HU210 (30 μg/kg, IP). The locomotor activity was quantified by assessing the disruptions of infrared beams in two dimensions for 4 h.

### Cataleptic Behavior

Catalepsy was assayed using the bar test. Mice were taken out of their home cages, and their forepaws were placed on a horizontal bar measuring 0.5 cm in diameter, positioned 4 cm above the bench surface. Typically, vehicle-treated mice released the bar within 2 s. Cataleptic behavior was defined as the mice remaining motionless, grasping onto the bar, with an arbitrary cutoff of 30 s. The CB_1_R antagonists, including rimonabant at doses 1 and 10 mg/kg and BNS808 at a dose of 1 mg/kg, were administered 30 min before the IP injection of 3 mg/kg WIN-55,212 (a CB_1_R agonist). The test was conducted 60 min after the administration of the agonist.

### Tissue Distribution of BNS808

Male C57Bl/6J mice aged 7–9 weeks (3–4 mice per compound) were fasted overnight before administering each test compound. The compounds were accurately weighed and mixed with an appropriate vehicle to prepare a clear oral solution. The formulations were freshly prepared on the dosing day, and the mice were administered the compound via oral gavage within 4 h of formulation preparation. 1, 2, 4, and 8 h after compound administration, the mice were euthanized, and approximately 200 μL of blood was collected via cardiac puncture for plasma preparation. Brain and liver tissues were harvested and processed for analysis. Sample analysis was performed using LC-MS/MS, as described in detail in the SI.

Tissue distribution after chronic administration was conducted similarly, except tissue collection was performed 24 h after the last drug administration and analyzed in the same manner as previously described.

### Statistical Analysis

Values are expressed as the mean ± SEM. Unpaired Two-tailed Student’s *t* test was used to determine the differences between the two groups. Results in multiple groups were compared by One-way ANOVA followed by a one-sided Tukey test or *t* test using GraphPad Prism v8 for Windows (San Diego, CA). Significance was set at *P* < 0.05.

## Supporting Information Available

Supplemental experimental methods, figures, and tables that provide additional information supporting the synthesis, characterization, and evaluation of the BNS library (PDF)
Molecular formula strings (CSV)
