# Supplementary material for: Synthesis and Pharmacological Characterization of Novel Peripheral Cannabinoid-1 Receptor Blockers Based on a Tricyclic Scaffold
Source: J Med Chem. 2025 Apr 21;68(9):9431–45. doi: 10.1021/acs.jmedchem.4c03132 (PMC12067444; doi:10.1021/acs.jmedchem.4c03132)
Supplement: Supplementary file 1 — jm4c03132_si_001.pdf [file jm4c03132_si_001.pdf]

Supporting Information

Synthesis and Pharmacological Characterization of Novel Peripheral Cannabinoid-1 Receptor Blockers Based on a Tricyclic Scaffold

Asaad Gammal[a, b, #], Taher Nassar[b, #], Yael Soae[c], Noam Freeman[c], Amit Badihi[c], Saja Baraghithy[a], Alina Nemirovski[a], Joseph Tam[a,*], and Simon Benita[b,*]

[a] Obesity and Metabolism Laboratory, The Institute for Drug Research, School of Pharmacy, Faculty of Medicine, The Hebrew University of Jerusalem, Jerusalem 9112001, Israel

[b] Laboratory of Nano Delivery Systems, The Institute for Drug Research, School of Pharmacy, Faculty of Medicine, The Hebrew University of Jerusalem, Jerusalem 9112001, Israel

[c] BioNanoSim (BNS), Hadassah Ein Kerem Campus, Minrav Building (JBP), Jerusalem, 9112101, Israel

*E-mail: yossi.tam@mail.huji.ac.il; Tel.: +972-2-675-7645

*E-mail: simonb@ekmd.huji.ac.il; Tel: +972-2-675-8668

[#]*A.G. and T.N. contributed equally to this work*

Table of Content

Supplementary Experimental Methods 3

Brain membrane preparation for CB_1R binding. 3

CB_1R and CB_2R binding. 3

Tissue preparation for [^{35}S]GTPγS binding. 5

[^{35}S]GTPγS binding - CB_1R activity. 6

Bidirectional permeability assessment in MDR1-MDCK cells. 6

Measurements of BNS808 using LC-MS/MS. 7

Supplementary Table 1. The collision energy (CE), declustering potential (DP), and collision cell exit potential (CXP) for the monitored transitions. 9

Mini Ames assay. 9

Protein binding of test compound in mouse brain using equilibrium dialysis method. 10

hERG assay. 12

Cell viability assay. 13

Supplementary Table 2. Summary of in vitro MDR-MDCKII permeability of BNS8 derivatives. 14

Supplementary Table 3. Mini Ames Study 15

Supplementary Table 4 – Mini Ames Study 17

Supplementary Table 5. Cell viability assay in HepG2 cells at 72 h and mutagenic effect in a Mini Ames assay. 19

Supplementary Table 6. The IC50 of BNS8 derivative compounds on whole cell hERG currents. 20

Supplementary Table 7. Stability of the test compounds in simulated gastric fluid (SGF). 21

Supplementary Table 8. Stability of the test compounds in simulated intestine fluid (SIF). 21

Supplementary Table 9. Protein binding of BNS808 in mouse brain, using equilibrium dialysis method. 22

Supplementary Figure 1. In vitro activity assessment of BNS compounds using the [^{35}S]GTPγS binding assay. 23

Supplementary Figure 2. BNS808 biodistribution and pharmacokinetics after chronic PO administration. 24

Supplementary Figure 3. The NMR, HPLC, and LC-MS analysis of BNS808 25

Supplementary Figure 4. The NMR, HPLC, and LC-MS analysis of BNS807 28

Supplementary Figure 5. The NMR, HPLC, and LC-MS analysis of BNS809 31

Supplementary Figure 6. The NMR, HPLC, and LC-MS analysis of BNS813 34

Supplementary Figure 7. The NMR, HPLC, and LC-MS analysis of BNS816 37

Supplementary Figure 8. The NMR, HPLC, and LC-MS analysis of BNS825 40

Supplementary Experimental Methods

Brain membrane preparation for CB_1R binding. The mouse cortex, excluding the cerebellum, was dissected from 6-week-old C57Bl/6Jhsd mice (Harlan, Israel). The tissue was homogenized in 4 mL Buffer 1 (320 mM sucrose, 2 mM EDTA, 5 mM $MgCl_2$) using a glass-Teflon homogenizer and Hei-TORQUE 200 (Heidolph; Cat# 17-012-002-25-1) homogenizer set to 132 rpm. After homogenization, the mixture was centrifuged at 1,600 × g for 10 min at four °C, and the resulting supernatant was kept on ice. The pellet (P1) was discarded, and the remaining supernatant was subjected to additional rounds of resuspension in 4 mL Buffer 1 followed by centrifugations, repeating this process three times. The pellets obtained from these centrifugations were discarded, and the three supernatant fractions were combined and subjected to higher-speed centrifugation at 39,000 × g for 15 min at four °C. The resulting supernatant was discarded, and the pellet (P2) was resuspended in 5 mL Buffer 2 containing 50 mM TRIS HCl pH 7.0, 0.2 mM EDTA, and five mM $MgCl_2$. This resuspended pellet (P2) was incubated at 37 °C for 10 min and then centrifuged at 23,000 × g for 10 min at 4°C. After centrifugation, the pellet (P2) was resuspended in 5 mL Buffer 2 and further incubated at 30 °C for 40 min. Following this incubation, the suspension was centrifuged at 11,000 × g for 15 min at 4 °C. The pellet (P2) was resuspended in 2 mL Buffer 3 containing 50 mM TRIS HCl pH 7.4, 1 mM EDTA, and 3 mM $MgCl_2$. The protein concentration of the final suspension was determined using the BCA assay. Subsequently, the suspension was diluted to a final concentration of 2 mg/mL in Buffer 3, aliquoted on ice, and stored at -80 °C for further use.

CB_1R and CB_2R binding. Binding to the CB_1R/CB_2R was assessed using competition displacement assays with [^{3}H]CP-55,940 as the radioligand. Purified membranes from the mouse brain were utilized for CB_1R assessment (as described above). Purified

membranes from cells overexpressing human CB_2R (Charles River CAT# A618) were employed for CB_2R evaluation.

For mouse CB_1R binding assays, the novel compounds were dissolved in dimethyl sulfoxide (DMSO) to reach a final concentration of 10 mM. Each compound was then diluted in Buffer 3 (as described above) supplemented with 0.14% BSA, resulting in concentrations ranging from 10^{-4} M to 10^{-10} M. Triplicates of 100 μL of each concentration were dispensed into siliconized glass tubes. At the same time, 100 μL of Buffer 3 with 0.14% BSA was dispensed into three tubes as blanks. CB_1R brain membranes (20 μg) were added to each tube and diluted with 800 μL Buffer 3 with 0.14% BSA. Subsequently, 5 nM of [^{3}H]CP-55,940 (100 μL) was added to each tube. The tubes were sealed with parafilm and incubated with shaking (120 rpm) in a water bath at 30 °C for 1 hour. Following incubation, 2 mL of cold Buffer 4 (50 mM TRIS HCl pH 7.4, 1 mg/mL BSA) was added to each tube.

To harvest the content of each tube, a Brandel M-24R Vacuum Harvester was employed, with a wet WHATMAN GF/B filter paper (Fired) (one membrane for 12 tubes) placed in the harvester after being washed twice with Buffer 4. The filter membrane in the harvester absorbed the full content of each tube. Then, the filter membrane was washed six times with Buffer 4. Each filter-glass membrane tube was excised and transferred to a 20 mL glass tube containing a 5 mL ULTIMA GOLD scintillation cocktail. The glass tubes were subjected to shaking for one hour at 25 °C and 200 rpm, following which they were read with a Tri-Carb 4910TR liquid scintillation counter.

Radioligand binding assays were performed to assess the binding affinity of the different compounds to hCB_1R (RBHCB1M400UA; PerkinElmer) or hCB_2R (RBXCB2M400UA; PerkinElmer). Competition displacement assays were employed,

utilizing [^{3}H]CP-55,940 as the radioligand and crude membranes from humans for CB_1R or CB_2R. The compounds were tested at various concentrations (eight points with a five-fold serial dilution starting at two mM), and their ability to displace [^{3}H]CP-55,940 was evaluated. Upon completion of the binding assays, the reaction mixture was filtered through GF/C plates, followed by four washes with cold wash buffer. After drying for one hour, 50 μL of Perkin Elmer Microscint 20 cocktail was added to the filter plate wells, and the bound [^{3}H]CP-55,940 radioligand was measured using a β counter. All data points were duplicated, and *Ki* values were determined from two independent experiments using GraphPad Prism 7.02 analysis software, extracted through nonlinear regression analysis.

Tissue preparation for [^{35}S]GTPγS binding. The mouse cortex, excluding the cerebellum, from C57Bl/6Jhsd mice (Harlan, Israel) was dissected in 10 mL of centrifugation buffer containing 50 mM TRIS HCl pH 7.4, 1 mM EGTA and three mM $MgCl_2$. The tissue was homogenized using a glass-Teflon and Hei-TORQUE 200 homogenizer (Heidolph; Cat#17-012-002-25-1) set to 132 rpm. The resulting suspension underwent centrifugation at 48,000 × g for 20 min at four °C, and the supernatant was discarded. The pellet obtained was homogenized again and subjected to a second centrifugation under the same conditions. Subsequently, the resulting pellet was resuspended in 1 mL of assay buffer composed of 50 mM TRIS HCl pH 7.4, 0.2 mM EGTA, 9 mM $MgCl_2$, and 150 mM NaCl, followed by homogenization. The protein concentration of the suspension was determined using the BCA assay, and the suspension was diluted to a final concentration of 2 mg/mL in assay buffer. The prepared suspension was aliquoted on ice and stored at -80 °C for further use.

[^{35}S]GTPγS binding - CB_1R activity. The tested compounds were initially dissolved in DMSO to a final concentration of 10 mM and subsequently diluted in assay buffer containing 0.14% BSA to achieve concentrations ranging from 10^{-4} M to 10^{-10} M. Samples of each concentration (50 µL) were then dispensed into siliconized glass tubes, with three tubes allocated for each concentration.

For non-specific binding assessment, 50 µL of assay buffer was added to additional tubes; for basal binding assessment, 50 µL of 100 µM cold GTP was added to three glass tubes. Furthermore, 50 µL of the solvent (assay buffer with 0.14% BSA) was dispensed in three extra tubes. Subsequently, 50 µL of 1 mM GDP was added to each tube, followed by ten µg CB1R (mouse) membranes to each tube. The volume was then adjusted to 350 µL with an assay buffer containing 0.14% BSA. Finally, 50 µL of 0.5 nM [^{35}S]GTPγS was added to each tube.

The glass tubes were covered with parafilm and incubated for one hour at 30 °C and 120 rpm. After one hour of incubation, the tubes were removed from the bath, and 2 mL of cold wash buffer composed of 50 mM TRIS HCl pH 7.4, 5 mM $MgCl_2$, and 1 mg/mL BSA was added to each tube. The content of each tube was harvested, allowing it to be absorbed into a wetted WHATMAN GF/B filter paper placed in the vacuum Brandel M-24R Harvester. The WHATMAN GF/B membrane was washed three times, cut, and transferred to a 20 mL glass tube with 5 mL of ULTIMA GOLD scintillation cocktail. The glass tubes were shaken at 25 °C for 1 hour at 200 rpm, and a Tri-Carb 4910TR liquid scintillation counter was used to read the radioactivity.

Bidirectional permeability assessment in MDR1-MDCK cells. Test and reference compounds were quantified using LC-MS/MS analysis based on the peak area ratio of analyte to internal standard (IS). The apparent permeability coefficient (Papp) in cm/s was computed using the equation: $\mathbf{P_{app} = (dC_r/dt) \times V_r / (A \times C_0)}$, where dC_r/dt

represents the cumulative concentration of the compound in the receiver chamber over time (μM/s), V_r is the solution volume in the receiver chamber (0.075 mL on the apical side, 0.25 mL on the basolateral side), A is the surface area for transport (0.0804 cm^2 for the monolayer area), and C_0 is the initial concentration in the donor chamber (μM).

The efflux ratio was determined using the equation **Efflux Ratio = P_{app} (B: A) / P_{app} (A: B)**. The percent recovery was calculated with the formula: **% Recovery = 100 × [(V_r × C_r) + (V_d × C_d)] / (V_d × C_0)**, where V_d represents the volume in the donor chambers (0.075 mL on the apical side, 0.25 mL on the basolateral side), and C_d and C_r are the final concentrations of transport compound in donor and receiver chambers, respectively.

Additionally, the percent of lucifer yellow in the basolateral well was calculated using the equation:

$$\textbf{Lucifer yellow} = \frac{\text{V basolatera} \times \text{R}FU\ basolatera}{\text{V apical} \times \text{RFU apical} + V\ basolatera \times RFU\ basolatera} \times 100$$

where RFU_{Apical} and $RFU_{Basolateral}$ demote the relative fluorescence unit values of lucifer yellow in the apical and basolateral wells, respectively, and V_{Apical} and $V_{Basolateral}$ represent the volumes of apical and basolateral wells (0.075 mL and 0.25 mL, respectively). The %Lucifer Yellow should be less than 2, indicating minimal leakage.

Measurements of BNS808 using LC-MS/MS. MS/MS analyses were conducted using a Sciex QTRAP® 6500+ mass spectrometer (Framingham, MA, USA). Air was generated using an SF4FF compressor (Atlas Copco, Belgium) and purified using an Infinity 1031 nitrogen generator (Peak Scientific, Inchinnan, Scotland). Purified air served as the source and exhaust gases, while purified nitrogen functioned as curtain and collision gases. A receiver was positioned between the compressor and the nitrogen generator to ensure a continuous and stable air supply.

Chromatographic separation was performed under reverse phase conditions using a Shimadzu UHPLC System (Kyoto, Japan), comprising a Nexera X2 LC-30AD pump, Shimadzu DGU-20A5R degasser, a Shimadzu SIL-30AC autosampler, and a Shimadzu CTO-20AC column oven. A CORTECS® column (C18, 2.7 µm particle size, 50 × 2.1 mm, Waters Corp., Milford, MA, USA) protected by a VanGuard® precolumn (Waters Corp., Milford, MA, USA) was employed for chromatographic separation. The autosampler was set at 15 °C, while the column was maintained at 40 °C throughout the analysis. Data acquisition was performed using Analyst 1.7.1 on a Dell Optiplex XE2 computer, and data analysis was conducted using Sciex OS Software. Gradient elution was achieved using mobile phases consisting of 0.1% formic acid (FA) in water (phase A) and 0.1% FA in acetonitrile (phase B). The gradient elution rate was set to 300 µL/min with an initial 5% B for the first 3 min, followed by a linear increase towards 65% B over 3 min, maintained at 65% B for 2 min, further increased linearly to 95% B in 0.5 min, and held at 95% B for 3 min.

BNS808 was analyzed in positive ion mode using electron spray ionization (ESI) and multiple reaction monitoring (MRM) acquisition modes, with BNS807 as the IS. The Turbo Spray IonDrive™ Turbo V source temperature was set at 400 °C, and the ion spray voltage was maintained at 3500 V. The curtain gas pressure was set at 35 psi. The nebulizer gas (Gas 1) was adjusted to 40 psi, while the turbo heater gas (Gas 2) was set to 50 psi. A dwell time of 30 msec was configured for the analysis. The collision energy (CE), declustering potential (DP), and collision cell exit potential (CXP) for the monitored transitions were determined and documented in **Supplementary Table 1**.

Supplementary Table 1. The collision energy (CE), declustering potential (DP), and collision cell exit potential (CXP) for the monitored transitions.

Compound	*Molecular ion [M+H]+ (m/z)*	*Fragment (m/z)*	*CE (volts)*	*DP (volts)*	*CXP (volts)*	*Retention time (min)*
		350 (quantifier)	30	1	18	
BNS808	550	250 (qualifier)	35	1	18	11.1
		357(quantifier)	25	1	26	
BNS807	528	111 (qualifier)	79	1	12	10.8

Mini Ames assay. To conduct the assay, top agar was melted with a microwave and maintained at a temperature ≥47 °C in a water bath. The assay was performed using a heat block set to 45 °C ± 2 °C. 12 × 75 mm test tubes were prepared in duplicates. For each concentration, the following components were added sequentially to a test tube: 1600 µL of top agar, 80 µL of the test compound or control substances, 400 µL of S9 mix (a liver enzyme mixture) or PBS buffer, and 80 µL of overnight culture. The mixture was vortexed and then dispensed into wells using a disposable pipette. The plates were then incubated at 37±2 °C for approximately 48 to 72 hours.

$$\mathbf{BoldBoldf} = \frac{\boldsymbol{Average\ number\ of\ revertants\ foe\ each\ dose\ group}}{\textbf{Average number of revertants for DMSO negative group}}$$

The mutagenicity of the compound was determined by evaluating the number of revertant colonies in strains TA98 and TA100. A compound was classified as mutagenic if the mean number of revertant colonies showed a dose-related and if the fold increase in revertants (F) was equal to or greater than 2 for both strains.

In addition, the cytotoxicity assessment included an evaluation of the background lawn growth on the agar plates. A scoring system ranging from 0 to 4 was employed for this assessment: 0 indicated no background lawn growth or complete cytotoxicity, while scores 1, 2, and 3 represented 25%, 50%, and 75% growth, respectively. A score of 4 signified full '100%' growth. The background lawn was evaluated using a stereoscope, with all observations compared to the vehicle control. The colonies appeared very small in plates with an entire background lawn and required magnification for visualization. In plates exhibiting toxicity, lawn colonies manifested as microcolonies (with a small diameter) or colonies (with a diameter similar to the revertant colony), which were visible without magnification.

Protein binding of test compound in mouse brain using equilibrium dialysis method. Solution A (0.5 mM) was prepared by mixing 10 µL of a 10 mM stock solution with 190 µL of DMSO. Subsequently, Solution B (0.02 mM) was prepared by adding 8 µL of solution A to 192 µL of 0.05 M sodium phosphate buffer. The final concentration of DMSO in Solution B was 4%.

A 96-well plate was initially prepared by dispensing 380 µL aliquots of brain tissue into wells designated for the brain while the buffer was added to the remaining wells to serve as a control. Subsequently, 20 µL of Solution B (containing 0.02 mM of test and reference compounds) was spiked into the pre-loaded brain samples in the plate. This step yielded a final test concentration of 1 µM, containing 0.2% DMSO. A duplicate system was established for the dialysis samples wherein brain tissue was prepared against buffer. Initially, 100 µL aliquots of blank dialysis buffer were applied to the receiver side of the dialysis chambers. Subsequently, 100 µL aliquots of the brain

tissue spiked with test and reference compounds were applied to the donor side of the dialysis chambers.

T0 brain samples for initial concentrations were prepared in duplicate. A 25µL aliquot of the brain spiked with test and reference compounds was transferred into a 96-well sample preparation plate. An equivalent volume of blank buffer was then added to each well. After the 5-hour incubation period, aliquots of 25 µL were taken from the dialysis apparatus's donor and receiver sides and transferred into new sample preparation plates. An equal volume of the opposite matrix (blank buffer to brain and vice versa) was added to each well, resulting in a final volume of 50 µL in each well. The samples (both T0 and five h) were then quenched with 200 µL of acetonitrile containing internal standard (IS) and vortexed at 600 rpm for 10 minutes. After vortexing, the samples were centrifuged at 5594 × g for 15 minutes. Subsequently, 50 µL of the supernatants were transferred to a new 96-well plate and mixed with 50 µL of Milli-Q water. The sample plates were covered and stored in a freezer (-20 °C) until LC/MS/MS analysis.

Warfarin and quinidine were used as moderate and high brain-protein binding, respectively. For data analysis, the following equations were used:

% Bound = 100 × ([Donor]5h -[Receiver]5h) / [Donor] 5h

% Recovery= 100 × ([Donor]5h +[Receiver] 5h) / [Initial] 0h

Fu (%) = 100 - %Bound

Undiluted Fu (%) = (1/D) / [(1/Fu2-1)+1/D]

Whereas; D: Dilution factor = Dilution factor of brain: 4

hERG assay. The electrophysiological assay was conducted with a holding potential of -80 mV. A brief voltage step to -50 mV was applied for 80 ms to eliminate any leak current. Subsequently, the voltage was stepped to +20 mV for 4,800 ms to induce the opening of hERG channels. After this activation step, the voltage was back down to -50 mV for 5,000 ms, resulting in a "rebound" or tail current, which was recorded for data analysis. Finally, the voltage was returned to the holding potential (-80 mV) for 1,000 ms.

For each experiment, a 40 μL volume of the vehicle (control) was added, and a baseline period of 300 sec was recorded. Then, doses of the test compounds were administered in 40 μL aliquots. The exposure of each concentration of the test compound lasted for no less than 300 sec. Five concentrations (0.30 μM, 1.00 μM, 3.00 μM, 10.00 μM, and 30.00 μM) were tested for each compound, with a minimum of 2 replicates per concentration. Data analysis was done using DataControl, Excel 2013 (Microsoft), and GraphPad Prism 5.0.

Within each recording well, the percent of control values was calculated for each test compound concentration based on peak current amplitude in the presence of reference control, using the formula: (current response/peak current) × 100%. Subsequently, dose-response curves were fitted to the standard Hill equation to determine the IC50 values using the equation:

$$\boldsymbol{I\text{post cpd} / I\text{pre cpd} = \text{Bottom} + (\text{Top-Bottom}) / [1+10^{(\text{LogIC50X})\times\text{HillSlope})}]}$$

where X is the logarithm of the concentration, *I*post cpd / *I*pre cpd is the normalized peak current amplitude, the Top is 1, and the Bottom is equal to 0. Curve fitting and IC50 calculations were performed using GraphPad Prism 5.0. If the inhibition obtained at the lowest concentration tested was over 50%, or at the highest

concentration tested was less than 50%, the IC50 was reported as less than the lowest concentration or higher than the highest concentration, respectively.

Cell viability assay. To assess the cell viability, which indicates the toxicity of the novel compounds, a total of 3,000 HepG2 cells were seeded into a 384-well plate. The compounds were then dispensed onto the cell plate using a Tecan D300e digital dispenser. Following compound addition, 35 μL CellTiter Glo reagent was added to all wells of the assay plate, and the plate was mixed for 30 sec on an orbital shaker to lyse cells. Subsequently, the plate was incubated at room temperature for 10 minutes. The luminescence signal was measured using a 384-well plate reader (EnVision). The obtained data were analyzed using XL-fit software (ID Business Solutions Ltd., Software version: XL fit 5.5.0.5). The percentage of inhibition was calculated using the formula:

Inhibition (%) = (1-Sample / ZPE) ×100

Sample represents the test compound's luminescence signal, and ZPE represents the luminescence signal from the zero-percent enzyme control.

Supplementary Table 2. Summary of in vitro MDR-MDCKII permeability of BNS8 derivatives.

ID#	GF120918 (+/-)	Mean Papp (10^{-6} cm/s)		Efflux Ratio	Mean recovery %		Rank	Note	P-gp substrate
		A to B	B to A		A to B	B to A	Papp		
**Nadolol**	-	0.42	ND	ND	101.89	ND	Low	Low permeability marker	-
**Metoprolol**	-	28.73	ND	ND	107.2	ND	High	High permeability marker	-
**Digoxin**	-	0.19	10.88	58.34	84.22	80.51	Low	P-gp substrate	**Yes**
	+	1.55	3.06	1.98	85.13	85.64			
**BNS808**	-	0.26	1.01	3.95	7.90	15.26	Low	*	**Yes**
	+	0.26	0.48	1.88	7.87	23.44			
**BNS815**	-	10.09	18.87	1.87	75.25	76.08	High	*	**Yes**
	+	15.82	9.96	0.63	63.83	77.57			
**BNS816**	-	6.99	5.37	0.77	41.56	48.84	High	*	**No**
	+	10.65	23.07	2.6	75.04	61.35			
**10q (BNS8)**	-	8.86	23.07	2.6	75.04	87.59	High	--	**Yes**
	+	20.08	13.91	0.69	63.83	86.10			

*The insufficient recovery (<50) might be caused by non-specific binding, cellular metabolism, cellular retention, or other issues

Supplementary Table 3. Mini Ames Study

TA98												
**Conditions**	**Compound ID**	**Concentration (µg/well)**	**Number of revertants**						**Growth of lawn**			**solubility**
			**Replicate 1**	**Replicate 2**	**Replicate 3**	**MEAN**	**SD**	**F value**	**Replicate 1**	**Replicate 2**	**Replicate 3**	
+S9	DMSO	0	9	13	11	11.00	2.00	1.00	4	4	4	P0
	BNS808	31.25	13	9	10	10.67	2.08	0.97	4	4	4	P0
		62.5	10	9	15	11.33	3.21	1.03	4	4	4	P0
		125	9	12	13	11.33	2.08	1.03	4	4	4	P0
		250	13	12	14	13.00	1.00	1.18	4	4	4	P1
		500	14	10	6	10.00	4.00	0.91	4	4	4	P2
		1000	8	10	11	9.67	1.53	0.88	4	4	4	P3
	2-aminoanthracene (2-AA)	2	362	320	364	348.67	24.85	31.70	4	4	4	P0
-S9	DMSO	0	7	6	9	7.33	1.53	1.00	4	4	4	P0
	BNS808	31.25	2	7	11	6.67	4.51	0.91	4	4	4	P0
		62.5	10	9	7	8.67	1.53	1.18	4	4	4	P0
		125	6	5	11	7.33	3.21	1.00	4	4	4	P0
		250	1	6	5	4.00	2.65	0.55	4	4	4	P1

		500	3	8	5	5.33	2.52	0.73	4	4	4	P2
		1000	3	2	5	3.33	1.53	0.45	4	4	4	P3
	2-nitrofluorene (2-NF)	4	112	92	104	102.67	10.07	14.00	4	4	4	P0

P0: no precipitations in solutions or growth of crystals on plates;

P1: 0~20% growth of crystals;

P2: 20%~60% growth of crystals;

P3: 60%~100% growth of crystals.

Supplementary Table 4 – Mini Ames Study
TA100

Conditions	Compound ID	Concentration (μg/well)	Number of revertants						Growth of lawn			solubility
			Replicate 1	Replicate 2	Replicate 3	MEAN	SD	F value	Replicate 1	Replicate 2	Replicate 3	
+S9	DMSO	0	40	34	45	39.67	5.51	1.00	4	4	4	P0
	BNS808	31.25	39	37	46	40.67	4.73	1.03	4	4	4	P0
		62.5	32	32	40	34.67	4.62	0.87	4	4	4	P0
		125	27	26	37	30.00	6.08	0.76	4	4	4	P0
		250	30	33	25	29.33	4.04	0.74	4	4	4	P1
		500	16	17	25	19.33	4.93	0.49	4	4	4	P2
		1000	16	13	21	16.67	4.04	0.42	4	4	4	P3
	2-aminoanthracene (2-AA)	2	316	324	312	317.33	6.11	8.00	4	4	4	P0
-S9	DMSO	0	28	35	27	30.00	4.36	1.00	4	4	4	P0
	BNS808	31.25	33	26	28	29.00	3.61	0.97	4	4	4	P0
		62.5	30	25	16	23.67	7.09	0.79	4	4	4	P0
		125	23	22	23	22.67	0.58	0.76	4	4	4	P0
		250	25	21	23	23.00	2.00	0.77	4	4	4	P1
		500	22	18	16	18.67	3.06	0.62	4	4	4	P2

		1000	19	11	16	15.33	4.04	0.51	4	4	4	P3
	Methylnitronitrosoguanidine (MNNG)	1	182	162	154	166.00	14.42	5.53	4	4	4	P0

P0: no precipitations in solutions or growth of crystals on plates;

P1: 0~20% growth of crystals;

P2: 20%~60% growth of crystals;

P3: 60%~100% growth of crystals.

Supplementary Table 5. Cell viability assay in HepG2 cells at 72 h and mutagenic effect in a Mini Ames assay.

ID#	Absolute IC_{50} (µM)	Relative IC_{50} (µM)	Top (%)	Bottom (%)	Slope	Ames assay
**BNS807**	>100	17.95	21.39	-12.17	1.52	ND
**BNS808**	26.2	16.84	62.98	-8.11	3.39	*Non mutagenic
**BNS815**	55.88	46.69	80.41	-9.25	3.71	ND
**BNS825**	>100	>100	6.27	-11.62	-0.21	ND
**Staurosporine**	0.08	0.06	71.33	1.13	2.53	ND

ND = not determined. *Detailed Results in the **Supplementary Tables 3-4**

Supplementary Table 6. The IC50 of BNS8 derivative compounds on whole cell hERG currents.

ID#	IC_{50} (μM)
**Amitriptyline**	3.49
**Amitriptyline**	2.58
**BNS807**	18.04
**BNS808**	5.39
**BNS815**	4.86

Supplementary Table 7. Stability of the test compounds in simulated gastric fluid (SGF).

ID#	Stability in SGF					
	Time (h)	With pepsin (mg)	Initial amount (%)	No pepsin (mg)	Initial amount (%)	Stability
**10q**	0	2.882	100	2.945	100	**Stable**
	1	2.879	100	2.968	101	
**BNS808**	0	2.753	100	2.826	100	**Stable**
	1	2.69	98	2874	102	

Supplementary Table 8. Stability of the test compounds in simulated intestine fluid (SIF).

ID#	Stability in SIF						
	Time (h)	With pancreatin (mg)	Initial amount (%)	Time (h)	No pancreatin (mg)	Initial amount (%)	Stability
**10 q**	0	3.035	100	0	2.77	100	Stable
	1	3.113	103	1	2.79	100	
	3	3121	103	3	3.572	101	
**BNS808**	0	2.972	100	0	3.257	100	Stable
	1	3.023	102	1	3.277	101	
	3	3.0009	101	3	3.266	100	

Supplementary Table 9. Protein binding of BNS808 in mouse brain, using equilibrium dialysis method.

**ID#**	**Species**	**Area ratio**			**Fraction of bound (%)**	**Fu (%)**	**Undiluted Bound (%)**	**Undiluted Fu (%)**	**Category**	**Recovery (%)**
		Donor 0h	Donor 5h	Receiver 5h						
**Warfarin**	Mouse brain	-	0.146	0.088	39.5	60.5	72.3	27.7	Moderate	-
**Quinidine**	Mouse brain	-	0.070	0.013	82.0	18	94.8	5.2	High	-
**BNS808**	Mouse brain	1.135	0.928	LOD	100	0.0	100.0	0.0	High	81.8

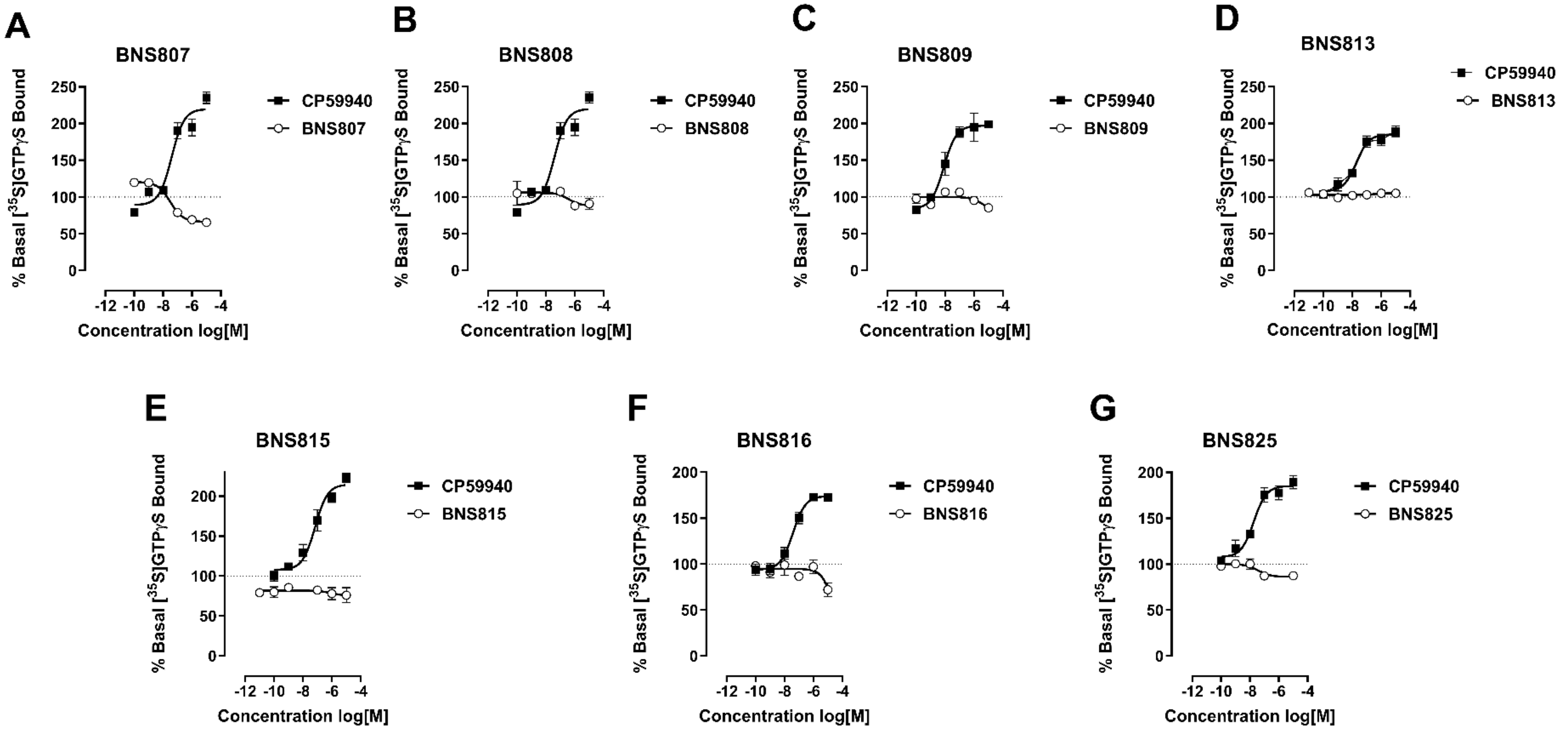

Supplementary Figure 1. In vitro activity assessment of BNS compounds using the [^{35}S]GTPγS binding assay. The assay was performed using mouse brain membrane preparations to evaluate CB_1R activity. CP-55,940, a synthetic cannabinoid receptor agonist, was used as a reference ligand to stimulate CB_1R, leading to G-protein activation. The tested compounds were evaluated for their ability to inhibit CB_1R-mediated activation (**A-G**). The results indicate that all compounds, except BNS807 (an inverse agonist), act as neutral antagonists, as demonstrated by their lack of basal activation.

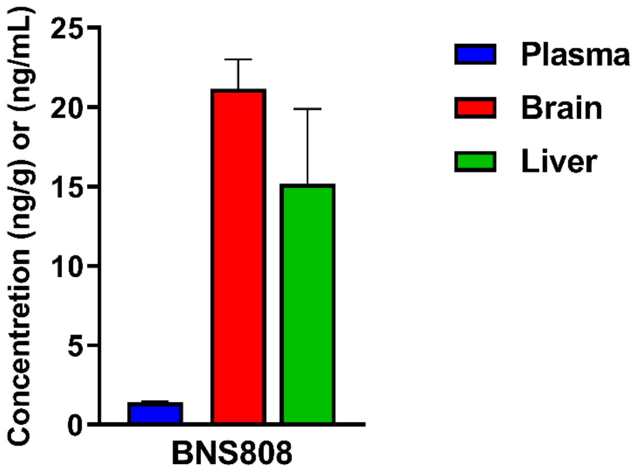

Supplementary Figure 2. BNS808 biodistribution and pharmacokinetics after chronic PO administration. The accumulation of BNS808 in organs/plasma was evaluated by analyzing the BNS808 levels in the plasma, brain, and liver post-chronic administration of 1 mg/kg PO for 24 days in diet-induced obese mice. Data represent the mean ± SEM of 4 mice per group.

Supplementary Figure 3. The NMR, HPLC, and LC-MS analysis of BNS808

<table>
<tr><td colspan="5">Compound Analysis</td></tr>
<tr><td rowspan="5">HPLC</td><td>Column</td><td colspan="3">Waters-XSelect, CSH-C18 2.5µm, 150mm×3mm</td></tr>
<tr><td>Method</td><td colspan="3">Gradient ACN: (H_2O + 0.1% FA)</td></tr>
<tr><td>Detection</td><td colspan="3">210nm</td></tr>
<tr><td>Purity</td><td colspan="3">99%</td></tr>
<tr><td>Notes</td><td colspan="3">LC peak at 8.772min corresponds to column and eluent.</td></tr>
<tr><td rowspan="2">LC-MS</td><td>Main mass peak</td><td>550</td><td>Expected exact mass</td><td>549</td></tr>
<tr><td>Source</td><td colspan="3">ESI</td></tr>
<tr><td rowspan="2">^{1}H NMR</td><td>Frequency</td><td>500 MHz</td><td>Solvent</td><td>$CDCl_3$</td></tr>
<tr><td>Notes</td><td colspan="3">Presence of traces of H_2O</td></tr>
</table>

^{1}H NMR

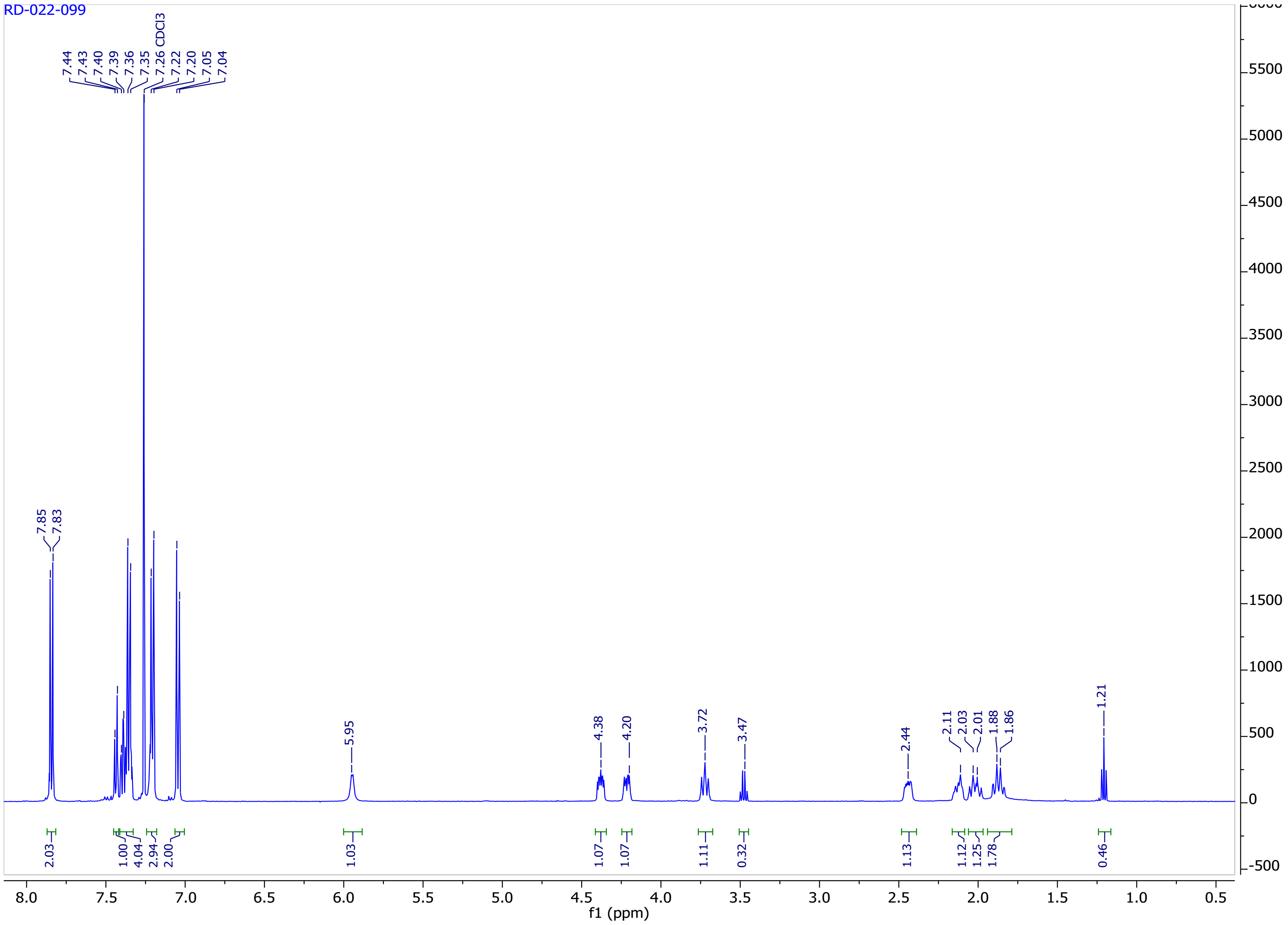
RD-022-099
7.85
7.83
7.44
7.43
7.40
7.39
7.36
7.35
7.26 CDCl3
7.22
7.20
7.05
7.04
5.95
4.38
4.20
3.72
3.47
2.44
2.11
2.03
2.01
1.88
1.86
1.21
2.03
1.00
4.04
2.94
2.00
1.03
1.07
1.07
1.11
0.32
1.13
1.12
1.25
1.78
0.46
f1 (ppm)

LC-MS

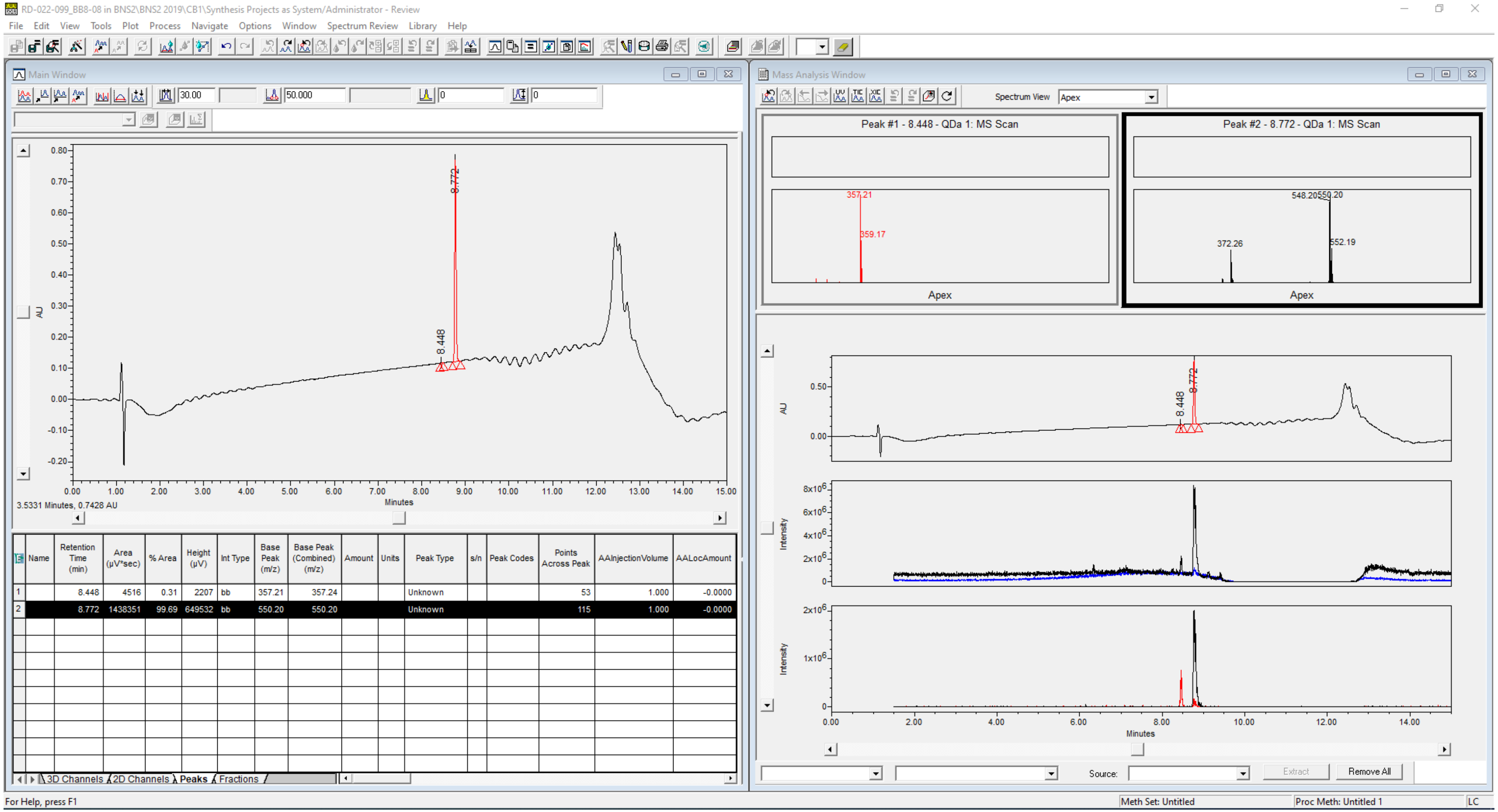
RD-022-099_BB8-08 in BNS2\BNS2 2019\CB1\Synthesis Projects as System/Administrator - Review
File Edit View Tools Plot Process Navigate Options Window Spectrum Review Library Help
Main Window
Mass Analysis Window
Spectrum View Apex
Peak #1 - 8.448 - QDa 1: MS Scan
357.21
359.17
Apex
Peak #2 - 8.772 - QDa 1: MS Scan
548.20550.20
372.26
552.19
Apex
8.772
8.448
AU
Minutes
Intensity
3.5331 Minutes, 0.7428 AU
	Name	Retention Time (min)	Area (µV*sec)	% Area	Height (µV)	Int Type	Base Peak (m/z)	Base Peak (Combined) (m/z)	Amount	Units	Peak Type	s/n	Peak Codes	Points Across Peak	AAInjectionVolume	AALocAmount
1		8.448	4516	0.31	2207	bb	357.21	357.24			Unknown			53	1.000	-0.0000
2		8.772	1438351	99.69	649532	bb	550.20	550.20			Unknown			115	1.000	-0.0000
3D Channels 2D Channels Peaks Fractions
Source:
Extract
Remove All
For Help, press F1
Meth Set: Untitled
Proc Meth: Untitled 1
LC

Supplementary Figure 4. The NMR, HPLC, and LC-MS analysis of BNS807

**Compound Analysis**				
HPLC	**Column**	Waters-XSelect, CSH-C18 2.5µm, 150mm×3mm		
	**Method**	Gradient ACN: (H_2O + 0.1% FA)		
	**Detection**	210nm		
	**Purity**	98%		
	**Notes**	LC peak at 8.508min corresponds to column and eluent.		
LC-MS	**Main mass peak**	528	**Expected exact mass**	528
	**Source**	ESI		
^{1}H NMR	**Frequency**	500 MHz	**Solvent**	$CDCl_3$
	**Notes**	Presence of traces of H_2O		

^{1}H NMR

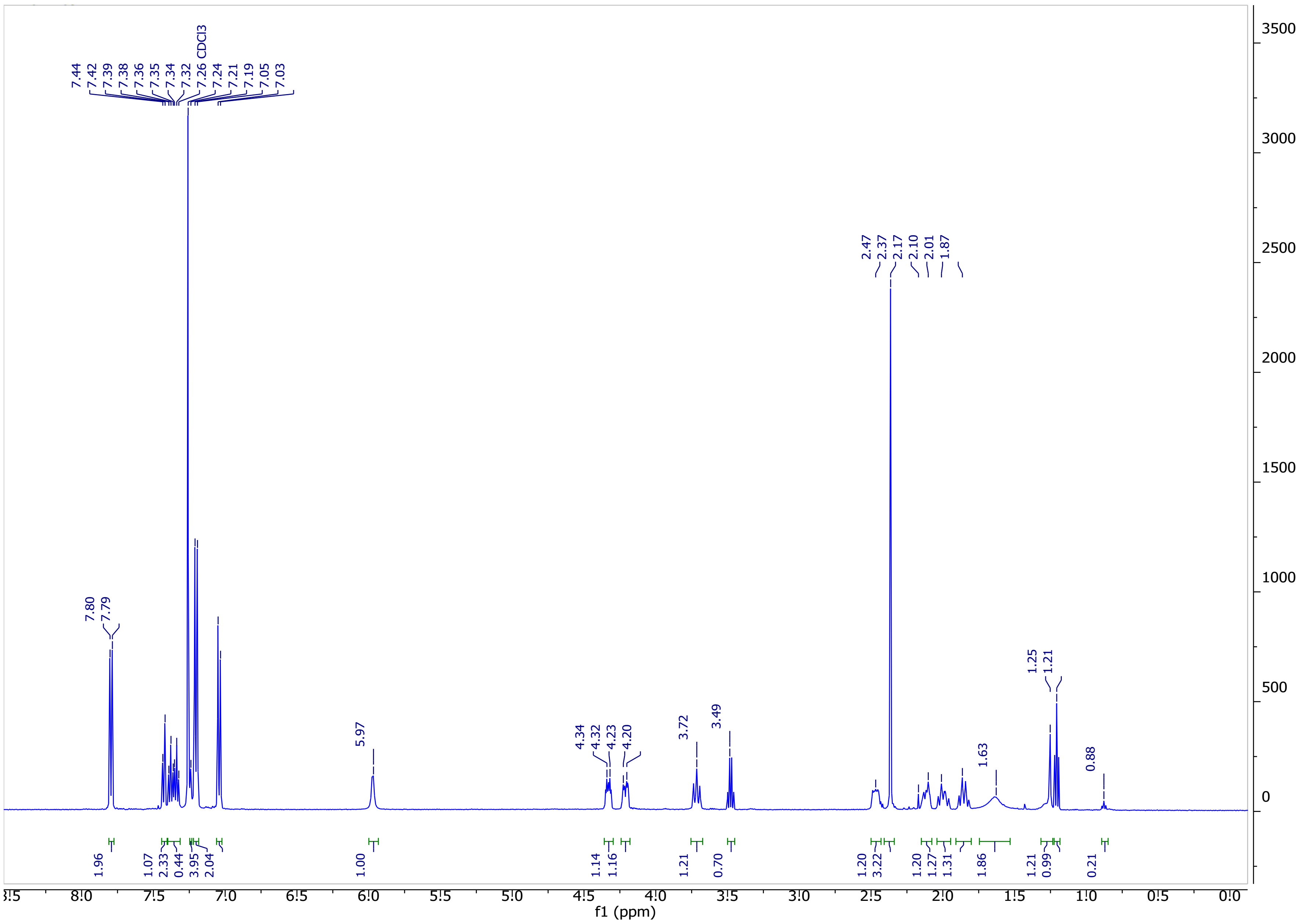
7.44
7.42
7.39
7.38
7.36
7.35
7.34
7.32
7.26 CDCl3
7.24
7.21
7.19
7.05
7.03
7.80
7.79
5.97
4.34
4.32
4.23
4.20
3.72
3.49
2.47
2.37
2.17
2.10
2.01
1.87
1.63
1.25
1.21
0.88
1.96
1.07
2.33
0.44
3.95
2.04
1.00
1.14
1.16
1.21
0.70
1.20
3.22
1.20
1.27
1.31
1.86
1.21
0.99
0.21
f1 (ppm)

LC-MS

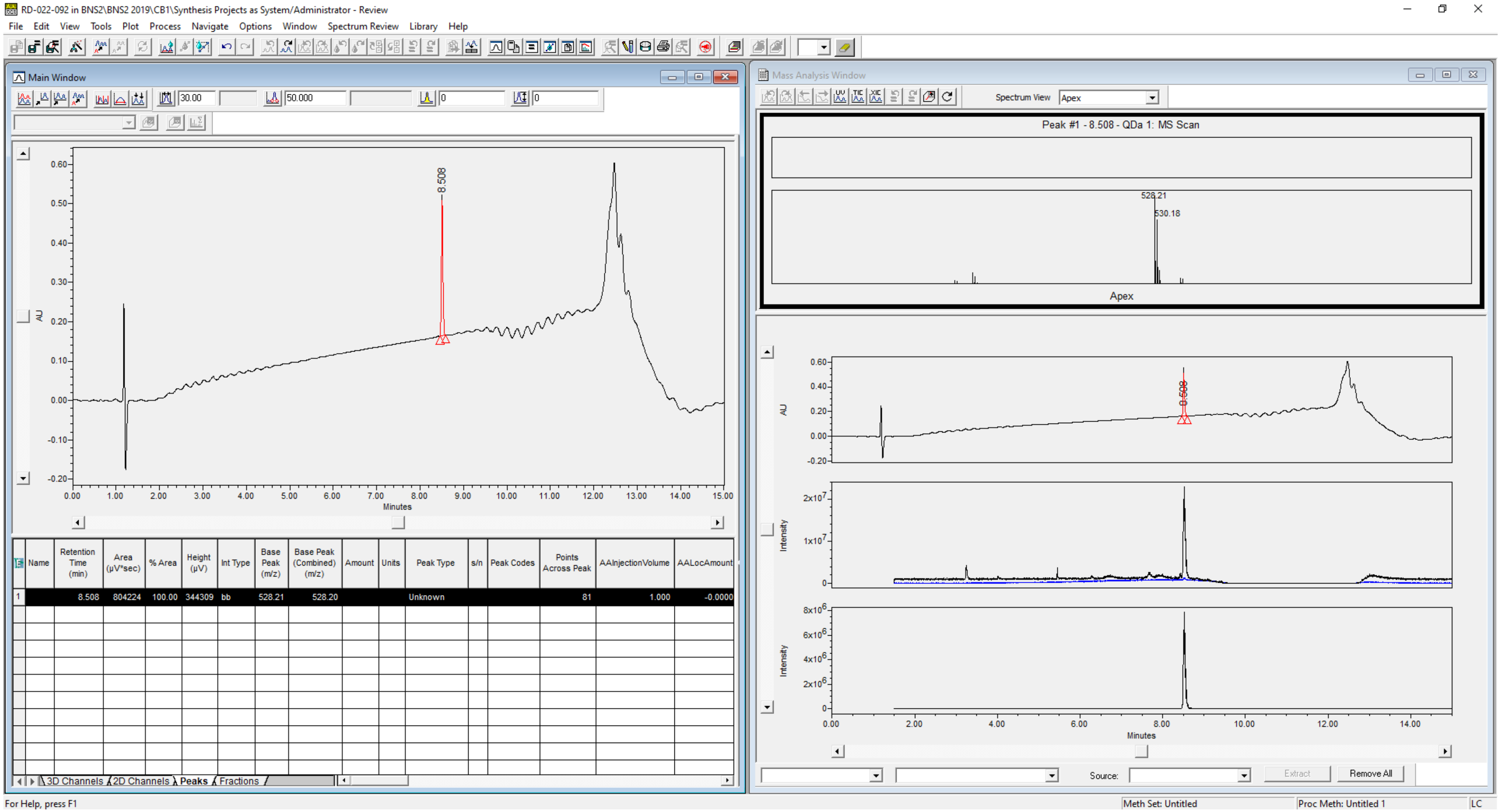
RD-022-092 in BNS2\BNS2 2019\CB1\Synthesis Projects as System/Administrator - Review
File Edit View Tools Plot Process Navigate Options Window Spectrum Review Library Help
Main Window
Mass Analysis Window
Spectrum View Apex
Peak #1 - 8.508 - QDa 1: MS Scan
528.21
530.18
Apex
8.508
AU
Minutes
Intensity
Name
Retention Time (min)
Area (µV*sec)
% Area
Height (µV)
Int Type
Base Peak (m/z)
Base Peak (Combined) (m/z)
Amount
Units
Peak Type
s/n
Peak Codes
Points Across Peak
AAInjectionVolume
AALocAmount
1 8.508 804224 100.00 344309 bb 528.21 528.20 Unknown 81 1.000 -0.0000
3D Channels 2D Channels Peaks Fractions
Source:
Extract
Remove All
For Help, press F1
Meth Set: Untitled
Proc Meth: Untitled 1
LC

Supplementary Figure 5. The NMR, HPLC, and LC-MS analysis of BNS809

**Compound Analysis**				
HPLC	**Column**	Waters-XSelect, CSH-C18 2.5μm, 150mm×3mm		
	**Method**	Gradient ACN: (H_2O + 0.1% FA)		
	**Detection**	210nm		
	**Purity**	97%		
	**Notes**	LC peak at 7.048min correspond to column and eluent.		
LC-MS	**Main mass peak**	452	**Expected exact mass**	452
	**Source**	ESI		
^{1}H NMR	**Frequency**	500 MHz	**Solvent**	$CDCl_3$
	**Notes**	Presence of traces H_2O		

^{1}H NMR

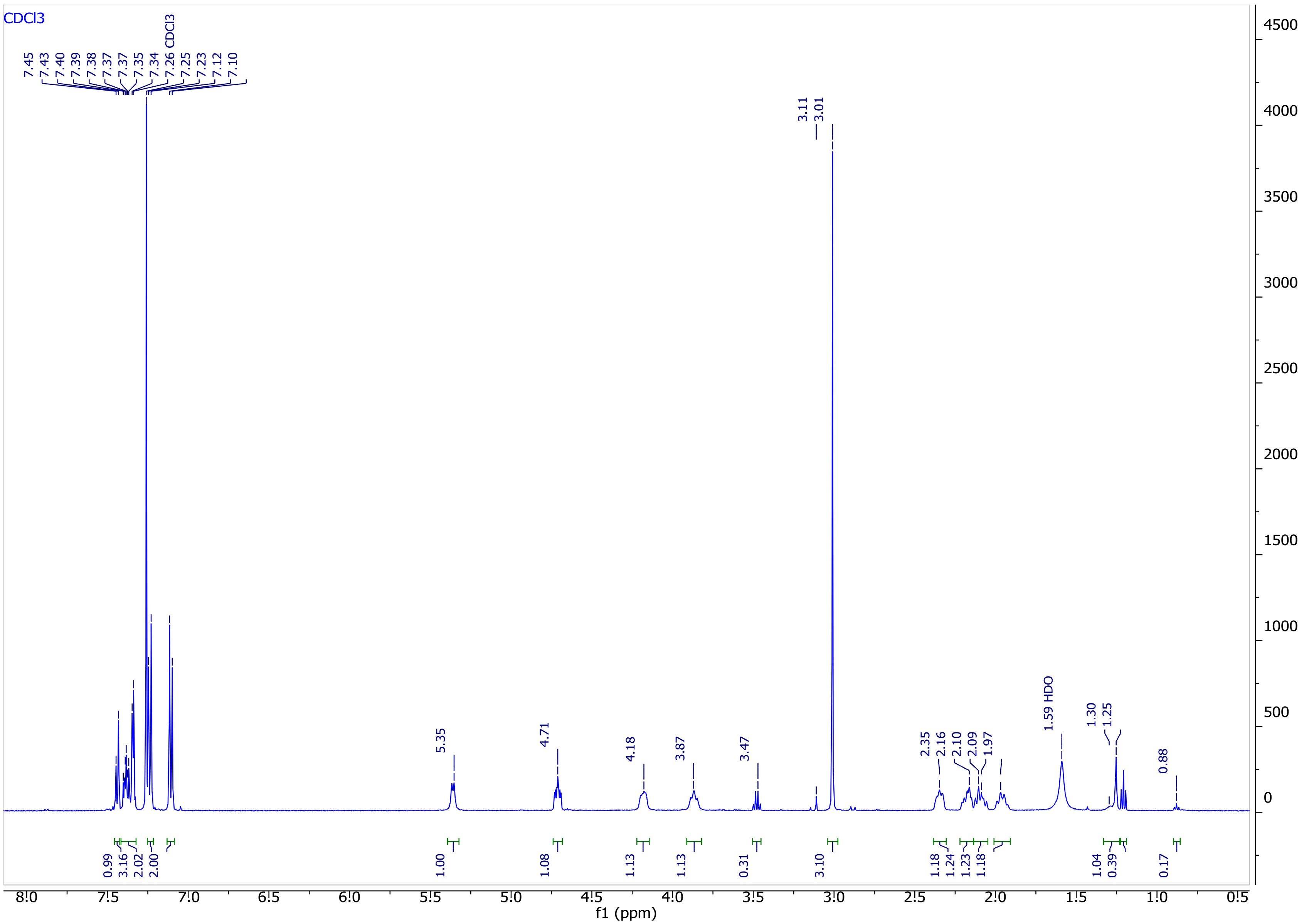

CDCl3
7.45
7.43
7.40
7.39
7.38
7.37
7.37
7.35
7.34
7.26 CDCl3
7.25
7.23
7.12
7.10
3.11
3.01
5.35
4.71
4.18
3.87
3.47
2.35
2.16
2.10
2.09
1.97
1.59 HDO
1.30
1.25
0.88
0.99
3.16
2.02
2.00
1.00
1.08
1.13
1.13
0.31
3.10
1.18
1.24
1.23
1.18
1.04
0.39
0.17
f1 (ppm)

LC-MS

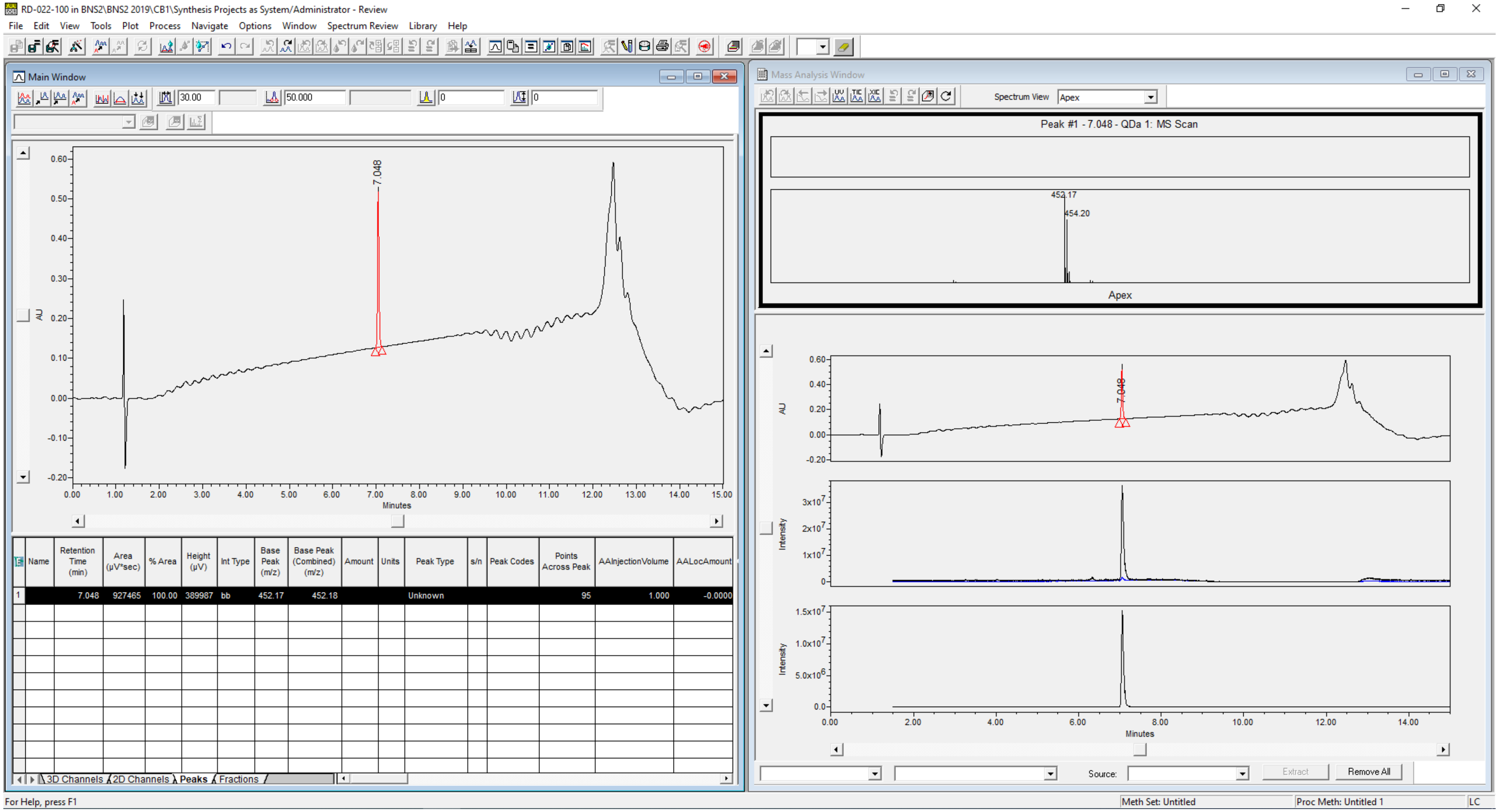

RD-022-100 in BNS2\BNS2 2019\CB1\Synthesis Projects as System/Administrator - Review
File Edit View Tools Plot Process Navigate Options Window Spectrum Review Library Help
Main Window
Mass Analysis Window
Spectrum View Apex
Peak #1 - 7.048 - QDa 1: MS Scan
452.17
454.20
Apex
7.048
AU
Minutes
Intensity
Name
Retention Time (min)
Area (µV*sec)
% Area
Height (µV)
Int Type
Base Peak (m/z)
Base Peak (Combined) (m/z)
Amount
Units
Peak Type
s/n
Peak Codes
Points Across Peak
AAInjectionVolume
AALocAmount
1 7.048 927465 100.00 389987 bb 452.17 452.18 Unknown 95 1.000 -0.0000
3D Channels
2D Channels
Peaks
Fractions
Source:
Extract
Remove All
For Help, press F1
Meth Set: Untitled
Proc Meth: Untitled 1
LC

Supplementary Figure 6. The NMR, HPLC, and LC-MS analysis of BNS813

**Compound Analysis**				
HPLC	**Column**	Waters-XSelect, CSH-C18 2.5μm, 150mm×3mm		
	**Method**	Gradient ACN: (H_2O + 0.1% FA)		
	**Detection**	210nm		
	**Purity**	97%		
	**Notes**	LC peak at 6.174min correspond to column and eluent.		
LC-MS	**Main mass peak**	431	**Expected exact mass**	431
	**Source**	ESI		
^{1}H NMR	**Frequency**	500 MHz	**Solvent**	$CDCl_3$
	**Notes**	Presence of traces H_2O		

^{1}H NMR

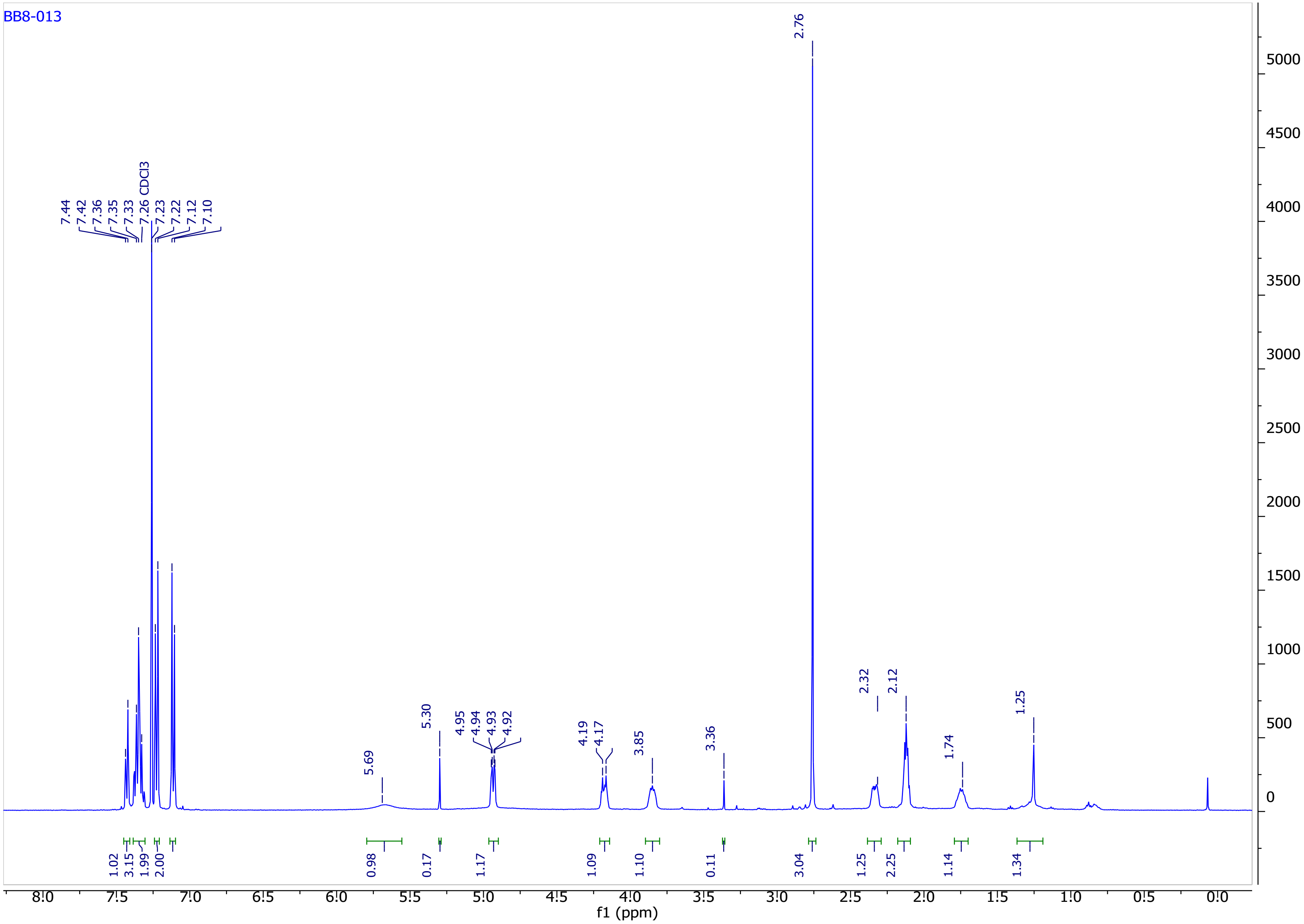

BB8-013
7.44
7.42
7.36
7.35
7.33
7.26 CDCl3
7.23
7.22
7.12
7.10
5.69
5.30
4.95
4.94
4.93
4.92
4.19
4.17
3.85
3.36
2.76
2.32
2.12
1.74
1.25
1.02
3.15
1.99
2.00
0.98
0.17
1.17
1.09
1.10
0.11
3.04
1.25
2.25
1.14
1.34
f1 (ppm)

LC-MS

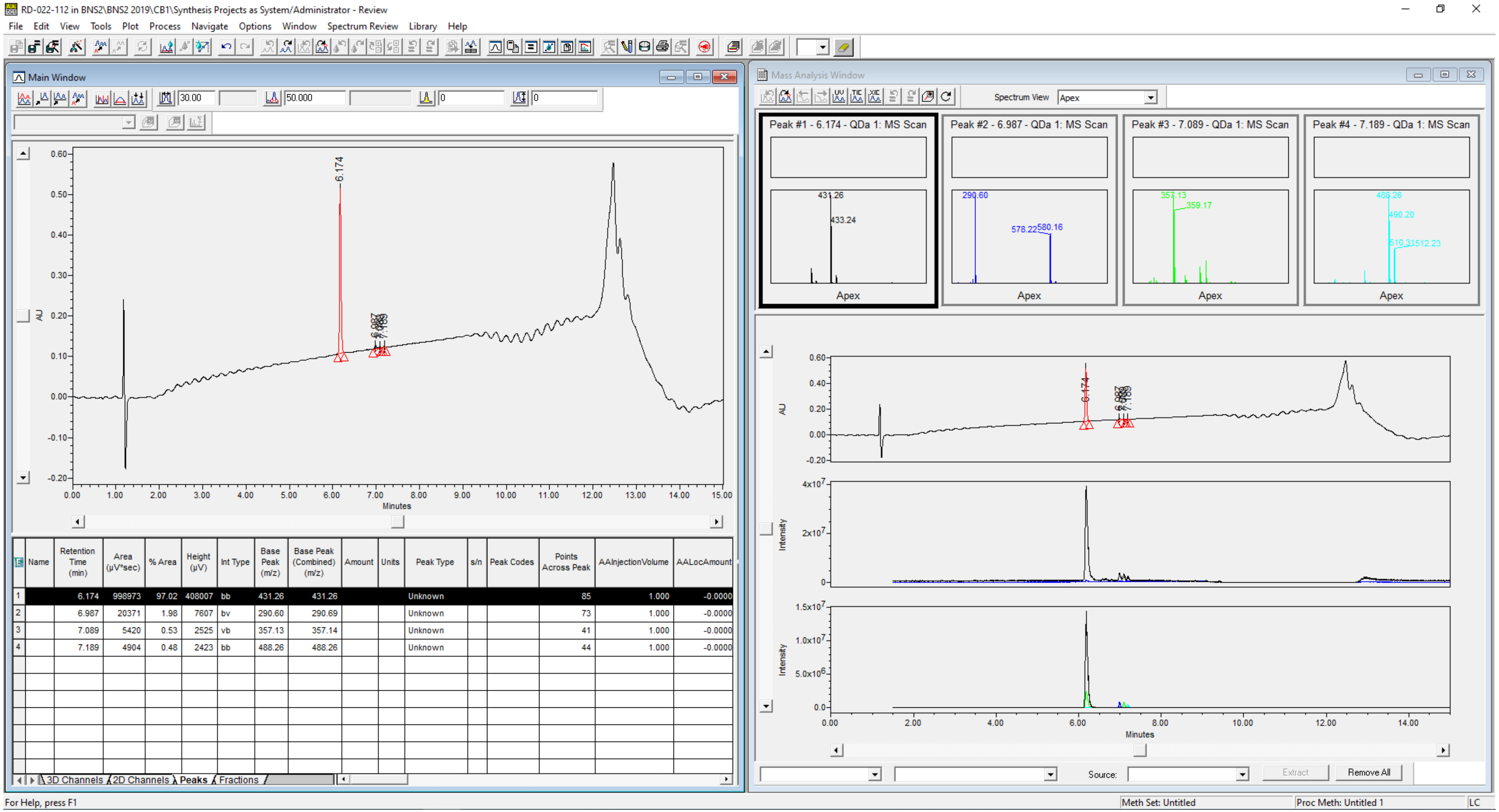

	Name	Retention Time (min)	Area (μV*sec)	% Area	Height (μV)	Int Type	Base Peak (m/z)	Base Peak (Combined) (m/z)	Amount	Units	Peak Type	s/n	Peak Codes	Points Across Peak	AAInjectionVolume	AALocAmount
1		6.174	998973	97.02	408007	bb	431.26	431.26			Unknown			85	1.000	-0.0000
2		6.987	20371	1.98	7607	bv	290.60	290.69			Unknown			73	1.000	-0.0000
3		7.089	5420	0.53	2525	vb	357.13	357.14			Unknown			41	1.000	-0.0000
4		7.189	4904	0.48	2423	bb	488.26	488.26			Unknown			44	1.000	-0.0000

Supplementary Figure 7. The NMR, HPLC, and LC-MS analysis of BNS816

**Compound Analysis**				
HPLC	**Column**	Waters-XSelect, CSH-C18 2.5µm, 150mm×3mm		
	**Method**	Gradient ACN: (H_2O + 0.1% FA)		
	**Detection**	210nm		
	**Purity**	98%		
	**Notes**	LC peak at 7.405min correspond to column and eluent.		
LC-MS	**Main mass peak**	557	**Expected exact mass**	556
	**Source**	ESI		
^{1}H NMR	**Frequency**	500 MHz	**Solvent**	DMSO
	**Notes**	Presence of traces H_2O		

^{1}H NMR

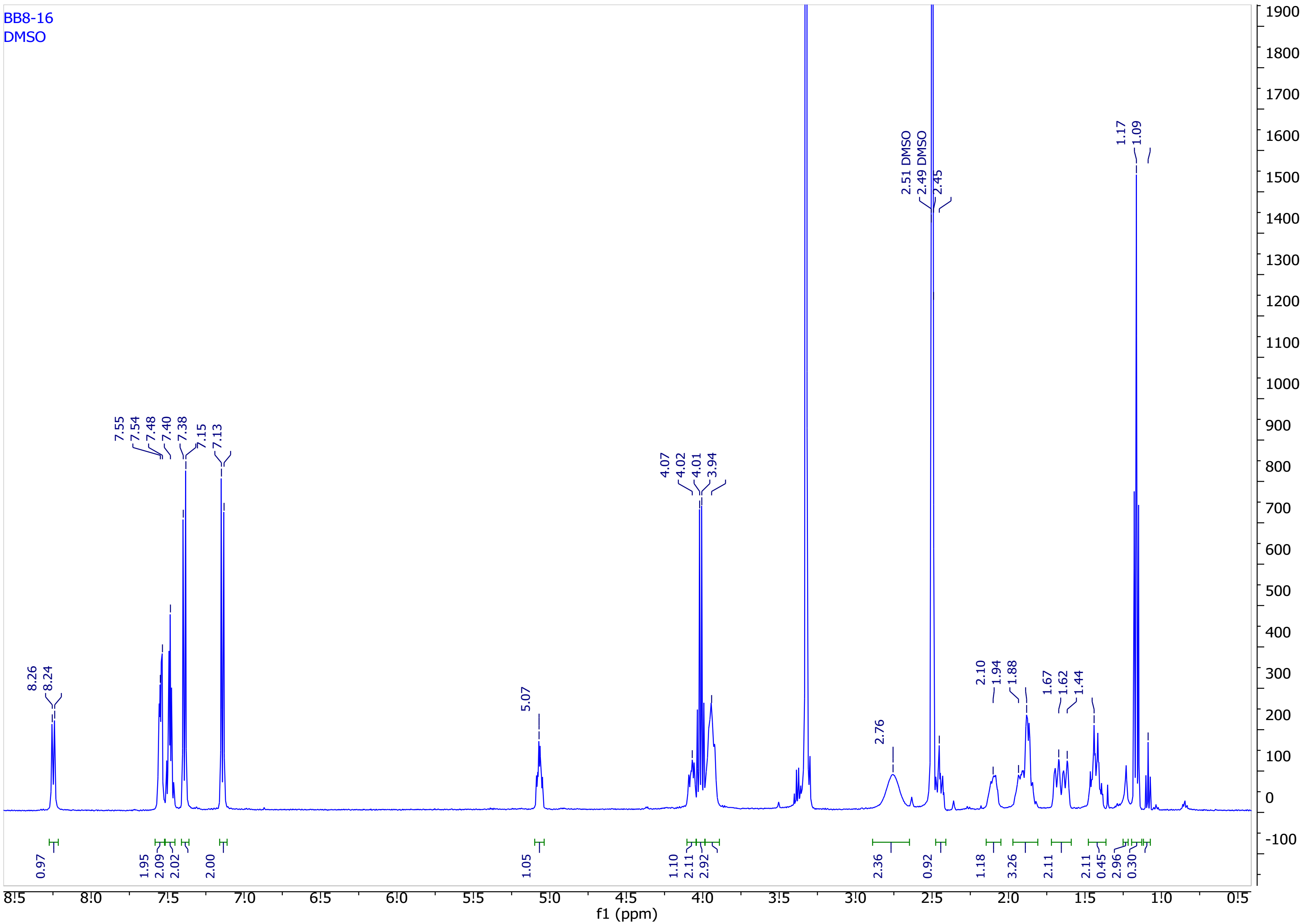

BB8-16
DMSO
8.26
8.24
7.55
7.54
7.48
7.40
7.38
7.15
7.13
5.07
4.07
4.02
4.01
3.94
2.76
2.51 DMSO
2.49 DMSO
2.45
2.10
1.94
1.88
1.67
1.62
1.44
1.17
1.09
0.97
1.95
2.09
2.02
2.00
1.05
1.10
2.11
2.92
2.36
0.92
1.18
3.26
2.11
2.11
0.45
2.96
0.30
f1 (ppm)

LC-MS

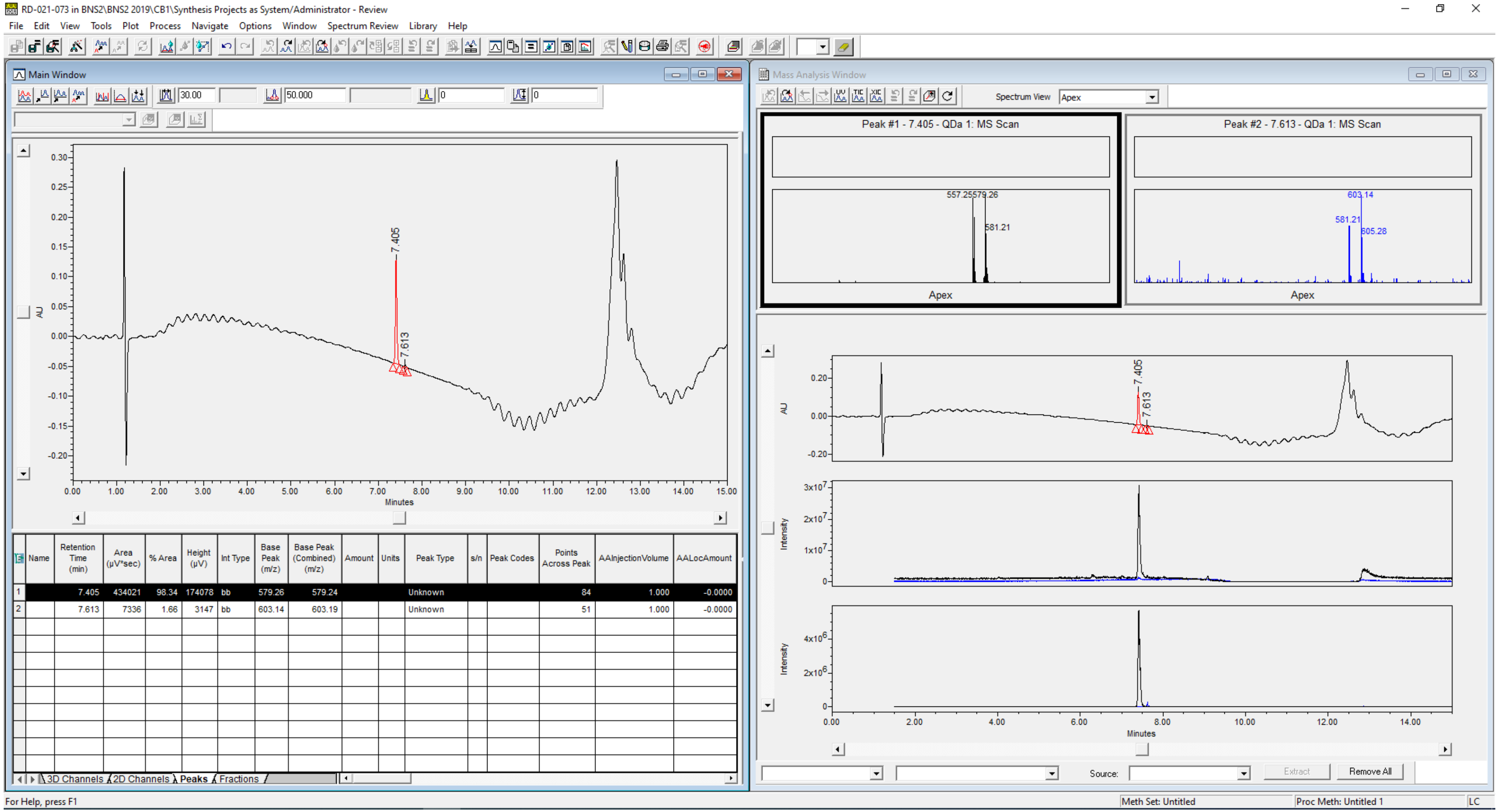

	Name	Retention Time (min)	Area (µV*sec)	% Area	Height (µV)	Int Type	Base Peak (m/z)	Base Peak (Combined) (m/z)	Amount	Units	Peak Type	s/n	Peak Codes	Points Across Peak	AAInjectionVolume	AALocAmount
1		7.405	434021	98.34	174078	bb	579.26	579.24			Unknown			84	1.000	-0.0000
2		7.613	7336	1.66	3147	bb	603.14	603.19			Unknown			51	1.000	-0.0000

Supplementary Figure 8. The NMR, HPLC, and LC-MS analysis of BNS825

**Compound Analysis**				
HPLC	**Column**	Waters-XSelect, CSH-C18 2.5µm, 150mm×3mm		
	**Method**	Gradient ACN: (H_2O + 0.1% FA), 15min		
	**Detection**	210nm		
	**Purity**	100%		
	**Notes**	LC peak at 7.849min correspond to column and eluent.		
LC-MS	**Main mass peak**	659	**Expected exact mass**	660
	**Source**	ESI		
^{1}H NMR	**Frequency**	500 MHz	**Solvent**	CDCl3
	**Notes**			

^{1}H NMR

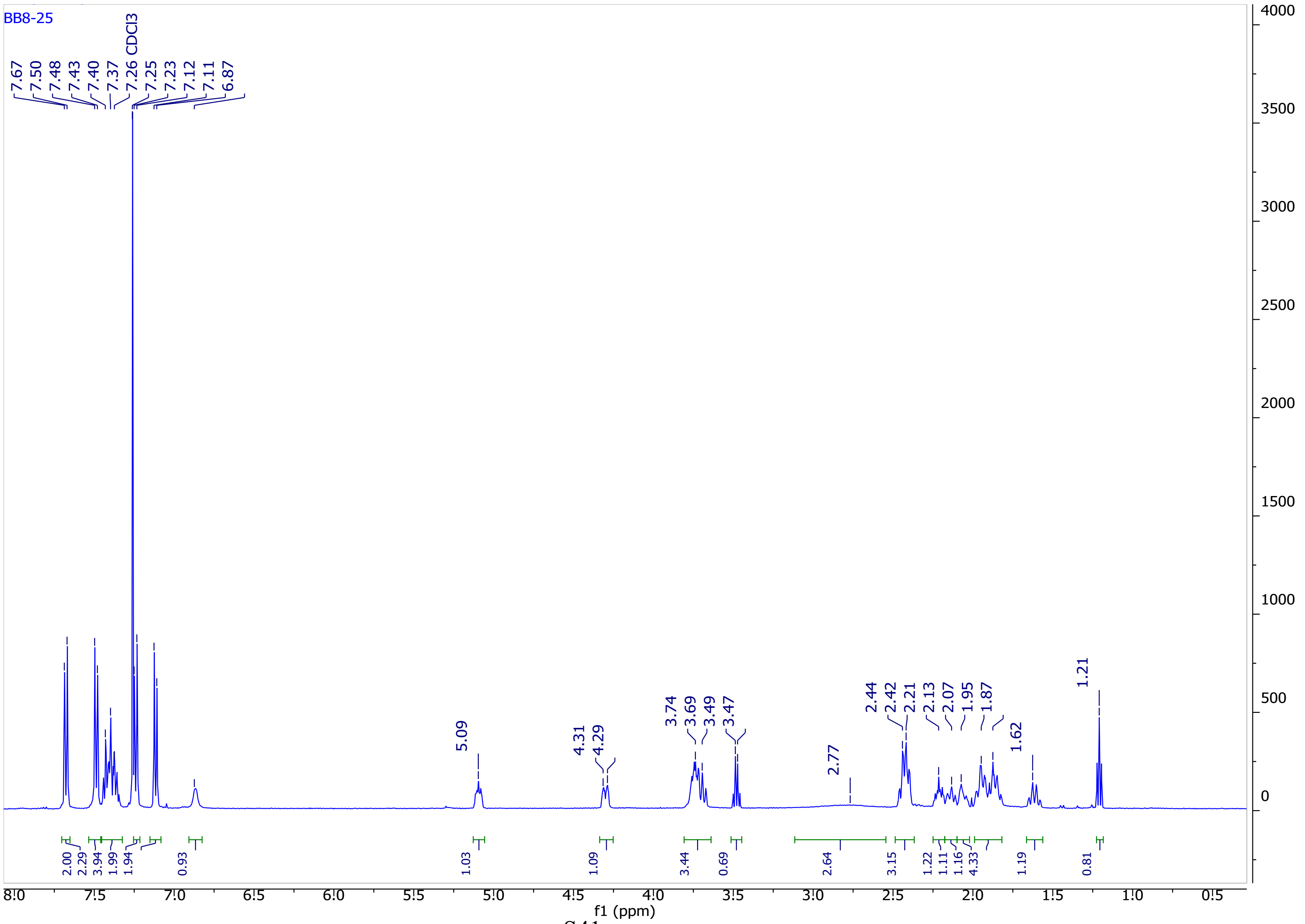

BB8-25
7.67
7.50
7.48
7.43
7.40
7.37
7.26 CDCl3
7.25
7.23
7.12
7.11
6.87
5.09
4.31
4.29
3.74
3.69
3.49
3.47
2.77
2.44
2.42
2.21
2.13
2.07
1.95
1.87
1.62
1.21
2.00
2.29
3.94
1.99
1.94
0.93
1.03
1.09
3.44
0.69
2.64
3.15
1.22
1.11
1.16
4.33
1.19
0.81
f1 (ppm)

LC-MS

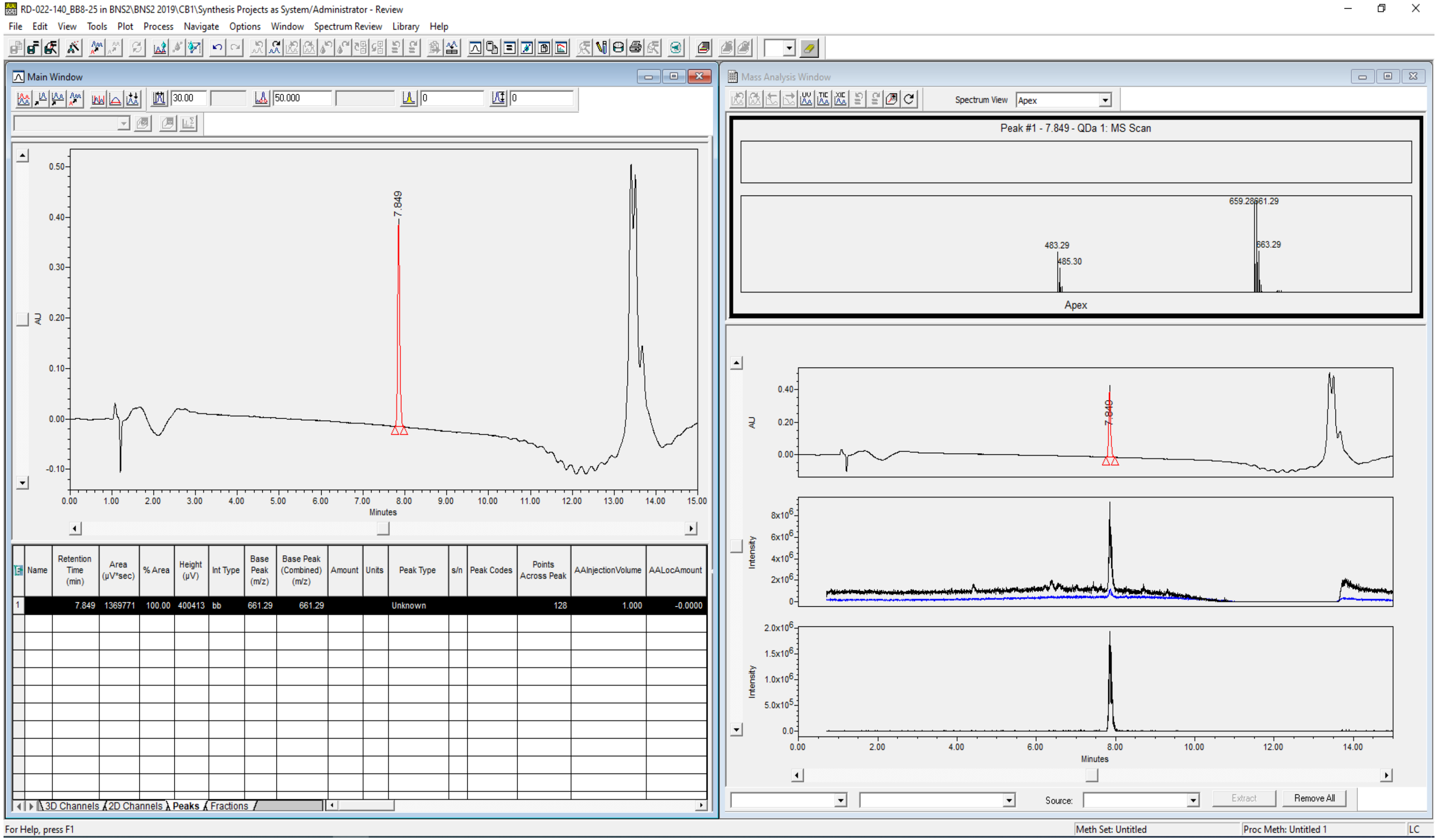

RD-022-140_BB8-25 in BNS2\BNS2 2019\CB1\Synthesis Projects as System/Administrator - Review
Main Window
Mass Analysis Window
Spectrum View Apex
Peak #1 - 7.849 - QDa 1: MS Scan
659.28661.29
483.29
485.30
663.29
Apex
7.849
AU
Minutes
Intensity
Name
Retention Time (min)
Area (µV*sec)
% Area
Height (µV)
Int Type
Base Peak (m/z)
Base Peak (Combined) (m/z)
Amount
Units
Peak Type
s/n
Peak Codes
Points Across Peak
AAInjectionVolume
AALocAmount
1 7.849 1369771 100.00 400413 bb 661.29 661.29 Unknown 128 1.000 -0.0000
For Help, press F1
Meth Set: Untitled
Proc Meth: Untitled 1
